# Simultaneous gene expression and multi-gene silencing in *Zea mays* using maize dwarf mosaic virus

**DOI:** 10.1186/s12870-021-02971-1

**Published:** 2021-05-05

**Authors:** Wenshuang Xie, Dee Marie Marty, Junhuan Xu, Nitika Khatri, Kristen Willie, Wanderson Bucker Moraes, Lucy R. Stewart

**Affiliations:** 1 Department of Plant Pathology, Ohio State University, OH 44691 Wooster, USA; 2 USDA-ARS Corn Soybean and Wheat Quality Research Unit, Wooster, OH 44691 USA

**Keywords:** Gene expression, Multigene silencing, Photobleaching, Potyvirus, Vascular puncture inoculation (VPI), Virus-induced gene silencing (VIGS)

## Abstract

**Background:**

Maize dwarf mosaic virus (MDMV), a member of the genus *Potyvirus*, infects maize and is non-persistently transmitted by aphids. Several plant viruses have been developed as tools for gene expression and gene silencing in plants. The capacity of MDMV for both gene expression and gene silencing were examined.

**Results:**

Infectious clones of an Ohio isolate of MDMV, MDMV OH5, were obtained, and engineered for gene expression only, and for simultaneous marker gene expression and virus-induced gene silencing (VIGS) of three endogenous maize target genes. Single gene expression in single insertion constructs and simultaneous expression of green fluorescent protein (GFP) and silencing of three maize genes in a double insertion construct was demonstrated. Constructs with GFP inserted in the N-terminus of HCPro were more stable than those with insertion at the N-terminus of CP in our study. Unexpectedly, the construct with two insertion sites also retained insertions at a higher rate than single-insertion constructs. Engineered MDMV expression and VIGS constructs were transmissible by aphids (*Rhopalosiphum padi*).

**Conclusions:**

These results demonstrate that MDMV-based vector can be used as a tool for simultaneous gene expression and multi-gene silencing in maize.

**Supplementary Information:**

The online version contains supplementary material available at 10.1186/s12870-021-02971-1.

## Background

Maize (*Zea mays*) is one of the most widely planted crops in the world, used for human consumption, animal feed, and as raw material for biofuels and other products (reviewed in [1]). In addition to its agricultural importance, maize is a model plant for monocot crop genetics, with complete genome sequences of B73 [2] and 34 other maize genomes deposited in Maize Genetics and Genomics Database (maizeGDB.org). However, few genomic tools for phenotyping impacts of individual genes are available. Maize transformation is technically difficult, expensive, and time consuming (reviewed in [3]). Thus, alternatives to transformation of stable transgenics are valuable. Virus-based tools developed for high-throughput virus-induced gene silencing (VIGS) could help fill the gap for maize gene function studies.

Many plant viruses have been developed as beneficial tools for expression of protein or VIGS (reviewed in [4–10]). VIGS has been used for gene functional analyses in host plants [11–14], utilizing target gene fragments in viral vectors in sense or antisense orientations or hairpin structures (see examples in [15]), with insertion sequence fragments as small as 100–300 nt being effective to silence target genes [13]. Plant viruses have also been widely used for protein expression in many different plant species (reviewed in [5, 16–18]). In one case, a plant virus vector derived from bean pod mottle virus (BPMV) was even developed for simultaneous single gene expression and single gene silencing [19]. For maize, several viruses have been developed as tools for gene expression or silencing, including brome mosaic virus (BMV) [20, 21], cucumber mosaic virus (CMV) [13], and maize rayado fino virus (MRFV) [22] for gene silencing [23], wheat streak mosaic virus (WSMV) [24–26] and sugarcane mosaic virus [27] for protein expression, foxtail mosaic virus (FoMV) for gene silencing [28] or protein expression [29] and guide RNA delivery for CRISPR/Cas9 gene editing [30], barley stripe mosaic virus for gene silencing [23] or protein expression [31] or for guide RNA delivery [32] and barley yellow striate mosaic virus (BYSMV) for simultaneous guide RNA and high cargo-capacity protein expression [33].

Viruses in the family *Potyviridae*, or potyviruses, have been developed as heterologous gene expression vectors in various plant hosts, enabling tracking of virus movement and other experiments. Viruses in the family *Potyviridae* have flexuous, rod-shaped virus particles with a single-stranded positive-sense RNA genome encapsidated by viral coat protein, and are the largest group of plant viruses (reviewed in [34]). The potyvirus genome encodes a polyprotein which is cleaved by its own viral proteases to produce 10 mature functional viral proteins for virus replication, pathogenicity, aphid transmission and other functions through interaction with many plant host proteins (reviewed in [34]). Potyviruses developed for protein expression include tobacco etch virus (TEV) [35, 36], zucchini yellow mosaic virus (ZYMV) [37], plum pox virus (PPV) [38], potato virus A (PVA) [39–41], turnip mosaic virus (TuMV) [42, 43], tobacco vein banding mosaic virus (TVBMV) [44], WSMV [24], and SCMV [27]. Because of their strong silencing suppressor, potyviruses were not favored for development of VIGS vectors, however, PVA was developed for transient gene silencing [45]. The most common insertion sites in these potyvirus-based vectors are the P1/HCPro and NIb/CP coding sequence junctions, with four other junction sites in the polyprotein open reading frame (ORF) or at the 5′ end of the polyprotein ORF utilized in some vectors.

Since more than one sequence insertion site can be utilized for some potyviruses, and their expandable rod-shaped virions are generally more tolerant of insertion sequences than viruses with icosahedral virions and inherent packaging constraints, expression of two to five proteins from a single vector has been achieved in some of these systems [36, 41–43].

Given the features of potyviruses and interest in further tools for maize, maize dwarf mosaic virus (MDMV) was an attractive option for development. MDMV was first reported in Ohio in 1963 [46]. MDMV is naturally transmitted by aphids in a nonpersistent manner [47], and is readily transmissible by rub inoculation in the laboratory [48]. MDMV infected plants have mosaic symptoms on leaves of susceptible cultivars of maize that can be visible as early as 5–7 days after rub inoculation. MDMV is common in the United States and is capable of reducing yields, but successful resistance breeding and disease management have limited major yield loss (reviewed in [49]). In addition to maize, the host range of MDMV includes some sorghum cultivars and Johnsongrass (*Sorghum halepense* L.), which is a major overwintering virus reservoir [50, 51]. Near-complete consensus sequence of a lab-maintained isolate MDMV OH2 (GenBank accession no. JQ403609) and complete sequence of a derived infectious clone (MDMV OH1; GenBank accession no. JQ403608) from Ohio were previously reported [52]. Here we report the cloning and sequencing of a new Ohio MDMV isolate from Johnsongrass, MDMV OH5, development of MDMV OH5 infectious clones, and engineered constructs for simultaneous gene expression and multi-gene silencing in maize. We report the first development of a virus-based vector for simultaneous gene expression and multi-target VIGS in maize.

## Results

### Complete sequence of MDMV OH5 and developing a full-length infectious clone

MDMV infection of Ohio-collected Johnsongrass (*S. halepense* L.) was confirmed by RT-PCR using previously published MDMV OH-specific primers to NIb (MDMV-7065F) and the 3’UTR (MDMV GenR1, [52], Additional file 1: Table S1), and by sequencing the amplified fragments. Typical mosaic symptoms developed when Johnsongrass leaf sap was rub-inoculated onto susceptible ‘Silver Queen’, ‘Early Sunglow’, and ‘Oh28’ maize plants (data not shown). RNA from original collected Johnsongrass leaves was used for cDNA synthesis, cloning and sequencing. The complete sequence of the MDMV isolate was determined by Sanger sequencing of amplicons, including 5' and 3' rapid amplification of cDNA ends amplicons (RACE; primers in Additional file 1: Table S1).

The precise 5′ sequence expressed from a cloned cDNA platform, and whether it is authentic, may be important or even essential in infectivity of the derived clones (see [53] and references therein). Thus, we experimentally determined the MDMV OH5 authentic 5′ most nucleotide using 5′-RACE as one of the first steps to generate optimal clones. The 5′-most nucleotide of MDMV OH5, identified by Sanger sequence of 5′ RACE amplicons, was adenosine, the same as for other published MDMV isolates: MDMV Bulgaria (GenBank accession no. NC_003377, [54], MDMV Golestan (GenBank accession no. JQ280313.1), MDMV Sz0605 (GenBank accession no. FM883211.2 [55], MDMV Mv0801 (GenBank accession no. FM883164.2 [55] (Table 1). RACE was also performed for MDMV-Italy (GenBank accession no. JX185302) and MDMV OH2, for which GenBank sequences were missing 5′-most sequences. This sequencing identified seven 5′ nucleotides for MDMV OH2 (5′-AAAAACA-3′) missing in the near-complete sequence submission and 35 5′ nucleotides missing in the near-complete sequence submission of MDMV Italy (5′-AAAAACAACAAGACTCAACACAACACAACCAAACA-3′). GenBank sequence submissions of near-complete sequences of these viruses were updated and completed with the 5′ sequence data for these viruses. Based on RACE data, all isolates had a 5′-most adenosine residue, rather than 5′-most guanine of the cloned virus MDMV OH1 (GenBank accession no. JQ403608), for which 5′- RACE on the MDMV OH2 source virus had not been successfully conducted [52]. All three MDMV OH sequences (MDMV OH1, MDMV OH2 and MDMV OH5) had the same 3′-end sequences as determined by 3′- RACE (Table 1). The complete sequence of MDMV OH5 was 9538 nt compared to the 9442 nt MDMV OH2 RACE-completed sequence, and shared 89% nucleotide and 96% polyprotein amino acid sequence identity with MDMV OH2. An additional 96 nucleotides present in MDMV OH5 were located in frame in the coding region for the N-terminus of the MDMV coat protein (CP) gene, such that MDMV OH5 is predicted to encode a coat protein containing 32 additional amino acids and with a predicted molecular weight of 34 kDa, compared to the 30 kDa CP predicted for MDMV OH1 and OH2. This additional sequence at the N-terminal CP coding sequence was previously found for all Ohio field isolates of MDMV in a survey of maize and Johnsongrass [56], suggesting that laboratory mechanically-passaged isolate (MDMV OH2) collected in Ohio ca. 1970 and the derived clone (MDMV OH1) may have lost N-terminal coat protein encoding sequence in passaging [56]. The complete sequence of the cloned Johnsongrass-derived field MDMV isolate was named MDMV OH5 and deposited as GenBank accession no. MN615724.

**Table 1:** 5′- and 3′-RACE of MDMV isolate genomic RNA

Virus isolate	GenBank accession no.	5′ RACE 5′ - 3 sequence	3′ RACE 5′ - 3 sequence
MDMV OH2	JQ403609.1	AAAAACAACAAGACT	TTCGTGGTGAGAGAC
MDMV It	JX185302.1	AAAAACAACAAGACT	TTCGTGGTGAGAGAC
MDMV OH5	MN615724	AAAAACAACAAGACT	TTCGTGGTGAGAGAC

**Fig. 1:**
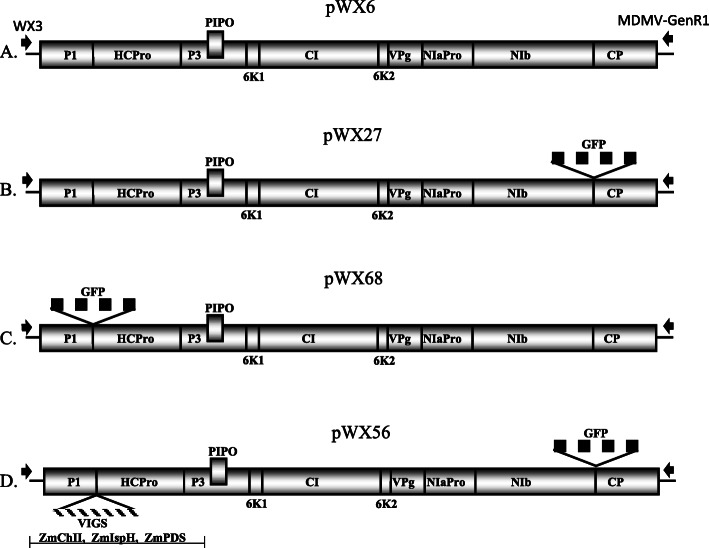
Genome layout of MDMV OH5 full-length infectious clone constructs. **a**. pWX6 (14,229 bp), infectious wild-type clone. **b**. pWX27 (15,009 bp), GFP gene inserted in-frame between NIb and CP coding sequences. **c**. pWX68 (15,018 bp), GFP gene inserted in-frame between P1 and HCPro coding sequences. **d**. pWX56 VIGS (15,798 bp), GFP gene inserted in-frame between NIb and CP coding sequences; and 249 bp each from target endogenous maize genes for magnesium chelatase (*Zm*ChII), lemon white 1 (*Zm*IspH), and phytoene desaturase (*Zm*PDS). Black arrows indicate location of 5′ WX3 and 3′ MDMV-GenR1 primers (Table S1) used for amplification prior to in vitro transcription. RNA transcripts were synthesized from amplicon with primer-added 5′ T7 promoter sequence. P1 = protein 1 protease, HCPro = helper component protease, P3 = protein 3 protease. PIPO = pretty interesting *Potyviridae* open reading frame. 6k_1_ = 6 kD protein 1. Cl = cylindrical inclusions. 6 k_2_ = 6kD protein 2. VPg = viral protein genome linked. NIa-Pro = nuclear inclusion a-protease. NIb- RdRP = nuclear inclusion b-RNA dependent RNA polymerase. CP = coat protein

**Fig. 2:**
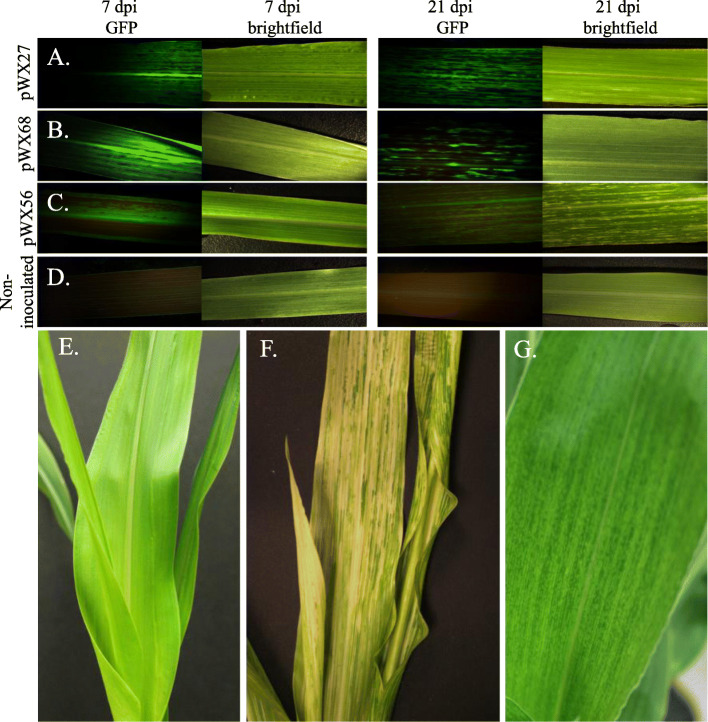
Images of GFP visualization and symptoms by MDMV OH5 constructs at 7 and 21-days post inoculation on *Z. mays* cultivar Silver Queen. **a**. pWX27. **b**. pWX68. **c**. pWX56. **d**. Non-inoculated control. **e**. Non-inoculated maize. **f**. pWX56-infected plant leaf with virus-induced gene silencing photobleaching. **g**. pWX6, a wild-type clone of MDMV OH5. The newest fully emerged leaf from top of the plants were selected for GFP visualization. **a-d**. Fluorescent images taken with a Leica DFC460C camera using fluorescence imaging with NIGHTSEA Green-only bandpass filter at 3 s exposure. Brightfield images using same camera without fluorescence at 1 s exposure. **e-g.** *Z. mays* ‘Silver Queen’ plant images at 21 dpi taken with a Canon EOS REBEL T5i

Full-length MDMV OH5 sequence was cloned into binary vector pJL89 [57] and named pWX6 (Fig. 1a). To examine infectivity of pWX6, in vitro RNA transcript derived from pWX6 was inoculated into ‘Silver Queen’ maize by vascular puncture inoculation (VPI [58, 59]) using amplified full-length sequences with primer-added T7 promoter to generate transcripts as was previously successful for the reported MDMV OH1 infectious clone [52]. Typical mosaic symptoms were observed in a subset of plants after inoculation (Fig. 2g), indicating that clone pWX6 is infectious with an infection rate of 33% by VPI (Table 2), an improvement over the ca. 10% maximum infection rates originally reported for the MDMV OH1 infectious clone [52]. Infection rates using this methodology were also improved for the original MDMV OH1 clone (57%), indicating that improvement was attributable to the modified inoculum preparation protocol (full-length sequence amplification and/or in vitro transcription, VPI procedure remained unchanged) rather than inherently higher infectivity of the MDMV OH5 pWX6 clone. Initial experiments to launch virus infection from the binary vector clones by agroinfiltration of maize or *Nicotiana benthamiana* from this construct were unsuccessful (data not shown), and further clones were tested using only the successful maize VPI approach.

**Table 2:** Efficiency of vascular puncture inoculation infection of MDMV OH5 derived constructs

	Rep 1	Rep 2	Rep 3	Rep 4	Rep 5	Rep 6	Rep 7	Rep 8	Rep 9	Total
**Clone**	**Infected/Inoculated**	**Infected/Inoculated**	**Infected/Inoculated**	**Infected/Inoculated**	**Infected/Inoculated**	**Infected/Inoculated**	**Infected/Inoculated**	**Infected/Inoculated**	**Infected/Inoculated**	**Infected/Inoculated**
**MDMV OH1**^**a**^	13/19	5/9	4/9	1/10	6/10	8/8	8/9	4/10	5/10	54/94 (57%)
**pWX6**	4/8	2/6	1/7	4/8	2/6	1/7	NT^b^	NT	NT	14/42 (33%)
**pWX27**	5/18	5/17	11/20	NT	NT	NT	NT	NT	NT	21/55 (38%)
**pWX68**	14/39	18/31	12/34	NT	NT	NT	NT	NT	NT	44/104 (42%)
**pWX56**	5/16	5/24	4/26	NT	NT	NT	NT	NT	NT	14/66 (21%)

^a^ Infectious clone reported in [52]

^b ^Not tested

### Expression of GFP from gene insertion at NIb/CP junction in pWX27

**Fig. 3:**
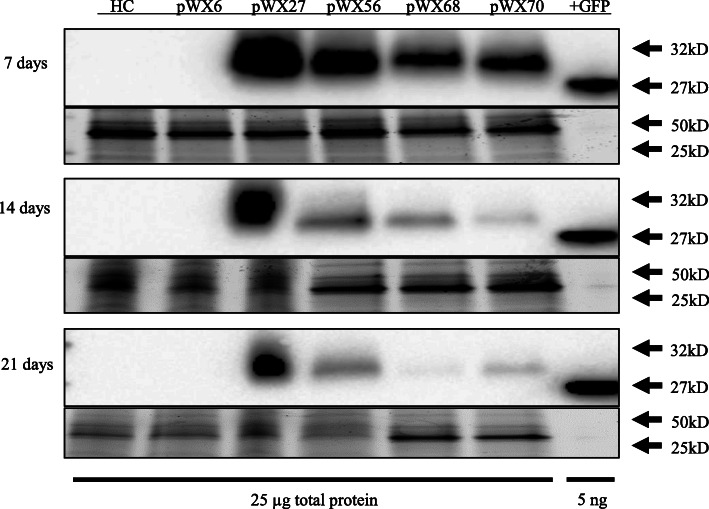
Western blot analysis of GFP in MDMV OH5 derived constructs. Samples of the newest fully emerged leaf from top of the plants were collected at 7, 14, and 21 days post rub-inoculation and analyzed for GFP protein using Anti-GFP antibody (Abcam, USA). Constructs were as diagrammed in Fig. 1, and pWX70 is similar to pWX56 with a similar-sized insert at P1/HCPro without further data reported. GFP from MDMV OH5 constructs with additional 6xHis and protease cleavage sequences with predicted molecular weight of 29.4 kDa were detected at slightly slower migration (32 kDa estimated based on ladder) compared to the positive control GFP (27 kDa estimated based on ladder) with predicted amino acid molecular weight of 26.8 kDa. Twenty-five μg total protein was loaded per sample and 5 ng recombinant *E. coli* GFP protein (Abcam, ab119740) was loaded as the positive control. Coomassie-stained gel loading control for each time point are shown in lower panels. Imaging of proteins was done on a Bio-Rad Imaging System utilizing Bio-Rad’s Stain Free Technology. Raw gel and blot images included in Additional file 3

To explore whether MDMV OH5 could be used for gene expression, the green fluorescent protein (GFP) gene was assembled in-frame within the polyprotein between nuclear inclusion b-RNA-dependent RNA polymerase (NIb-RdRP) and CP genes of MDMV OH5 in clone pWX6 to create pWX27 (Fig. 1b). NIb/CP cleavage sequence (EVIDVKHQAGE) was duplicated upstream and downstream of the inserted GFP sequence, with degenerate coding sequences to avoid direct sequence duplication, in order to allow efficient GFP cleavage from encoded polyprotein. Clone pWX27 was confirmed to be infectious with an average infection rate of 37% by VPI (Table 2), and typical mosaic symptoms developed in infected plants. GFP expression was visualized under a fluorescent microscope, indicating that MDMV OH5 could be used as a gene expression vector (Fig. 2a). Western blots for GFP showed that the amount of expressed GFP detected (expected size 29.4 kDa) declined slightly at weeks two and three compared to week one (Fig. 3; raw images shown in Additional file 3).

### Expression GFP from gene insertion at P1/HCPro junction in pWX68

We next inserted GFP between P1 and HCPro coding sequence in-frame within the polyprotein to create clone pWX68 (Fig. 1c). Clone pWX68 infectivity was confirmed by VPI with average infection rate of 43% (Table 2). Typical mosaic symptoms were observed, and GFP expression was visualized (Fig. 2b). Western blot analysis of pWX68-infected leaves also showed GFP expression (Fig. 3; raw images shown in Additional file 3).

### Simultaneous silencing of three genes and heterologous GFP expression from pWX56

Since GFP expression from gene insertion at each of two sites was successful, we next examined whether MDMV OH5 could be used for simultaneous gene silencing and gene expression using both insertion sites in a single construct. Using similar insertion sizes for VIGS, we aimed to target multiple genes in one insertion site. A triple gene-targeting VIGS insertion, *Zm*ChlI-IspH-PDS, was created to silence magnesium chelatase, lemon white 1 and phytoene desaturase genes, with 249 nt sequence from each targeted maize gene for a 747 nt total construct size. This triple-gene VIGS construct was inserted in-frame within the polyprotein between P1 and HCPro with inserted NIb cleavage site sequence of MDMV OH5 into a pWX27 backbone to create pWX56 (Fig. 1d). The infectivity of clone pWX56 was confirmed by VPI of RNA transcripts, with an average infection rate of 22% (Table 2).

**Fig. 4:**
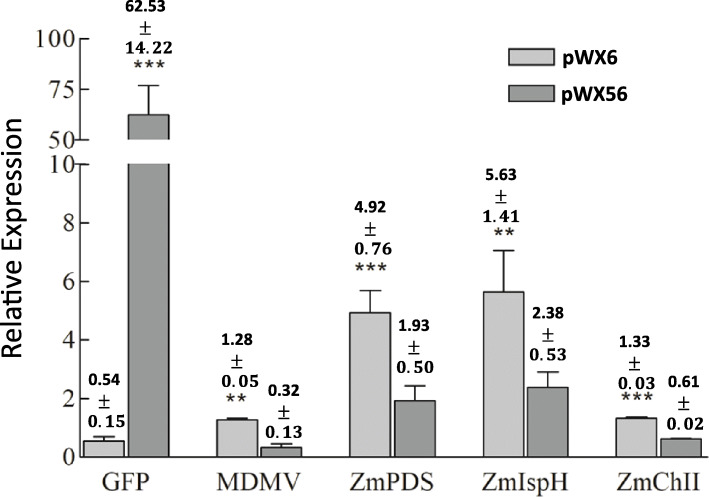
Mean target gene transcript relative expression ratios of 14 dpi of pWX56 inoculation. Mean values were derived from three samples of the newest fully emerged leaf of plants inoculated with wild type cloned virus (pWX6), and 6 samples of pWX56-infected plants. Folypolyglutamate synthase (FPGS) reference gene was used for all calculations (primers Table S1). Error bars represent standard error. Bars headed by asterisk * are significantly different with *p*-values <=0.05 (*), 0.01 (**) and 0.001 (***). Student's T-test was used

Green fluorescence was visualized in infected plants (Fig. 2a-d), and GFP protein expression confirmed by Western blot analyses (Fig. 3). Strong photobleaching was also observed in all leaves and stem tissue (Fig. 2FG, Fig. S1D) and tassels (data not shown). Silencing was not tested in root or other tissues. Retention of GFP coding sequences and sequences of each component of the triple VIGS insertion were verified by RT-PCR analysis (Additional file 2: Fig. S1A). Decreased levels of chlorophyll were measured compared to plants inoculated with the wild type clone pWX6 or healthy control plants (Additional file 2: Fig. S1B). Quantitative RT-PCR (RT-qPCR) was used to examine individual target gene transcript reduction of pWX56. All three genes targets, *Zm*ChlI, *Zm*PDS and *Zm*IspH, had reduced RNA transcript levels compared to wild type pWX6 infected plant controls at 14 days post inoculation (Fig. 4).

### Differential stability of constructs

**Fig. 5:**
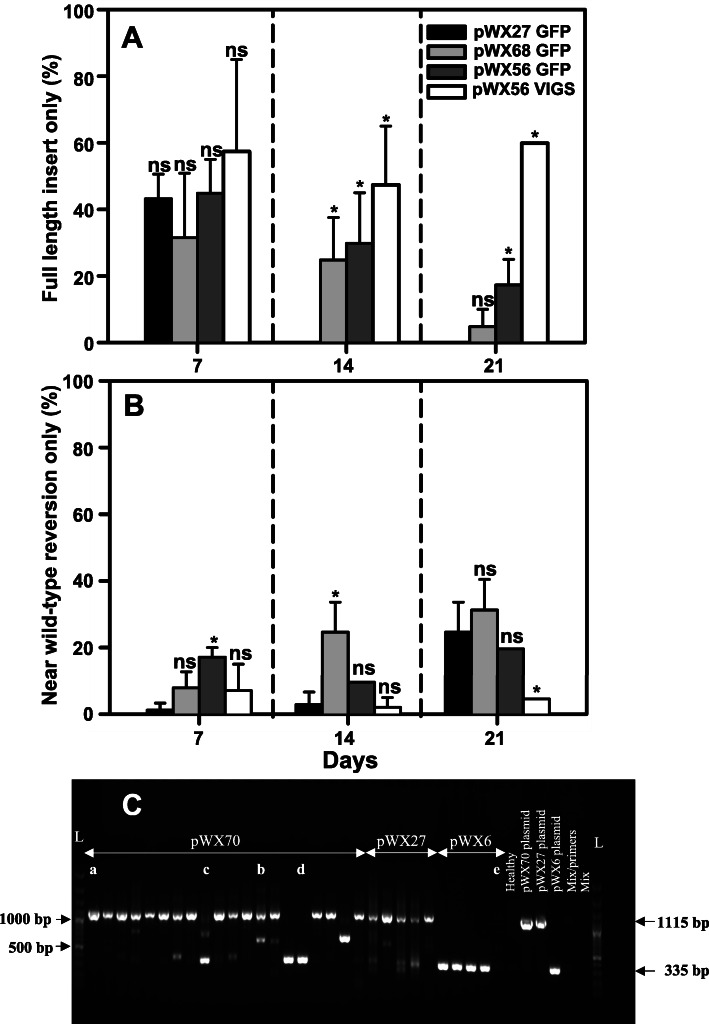
Comparison of insert stability in MDMV OH5 constructs at 7, 14, and 21 dpi. Across three replicates of 20 plants per construct, plants infected with pWX27, pWX68, and pWX56 were assessed for integrity of insertion sequences using RT-PCR with primers flanking the insertion sites. Five plants inoculated with pWX6, the cloned wild type virus, were included for each replicate. For each sample, amplicons were scored in five categories (examples from pWX70 shown in panel C); as full length insert **a**, full length insert plus smaller bands detected **b**, multiple bands with no full length insert **c**, single band of less than full-length insert usually near-wild type virus amplicon **d**, and no band detected **e** (example from pWX6 sample shown), compared to plasmid controls pWX70, pWX27 and pWX6 (WT). The number of samples in which bands were scored in categories **a** and **d** (panel A and B, respectively) were compared statistically for each construct. At fixed assessment times, constructs designated "ns" were not significantly different from pWX27 GFP at *P* ≤ 0.05 and constructs with * were significantly different from pWX27 GFP at *P* ≤ 0.05 based on the pairwise comparisons of least squares mean on the arcsine-square root scale. Samples were taken from the youngest leaves of 7, 14 and 21 dpi of plants for RT-PCR analysis using primers: WX112/WX111 (pWX27); WX317/WX315 (pWX68); WX358/WX367 for GFP insertion (pWX56), and primers WX317/WX315 for VIGS insertion (pWX56). Example test samples shown in panel C are from 14 dpi inoculations with pWX70; L = GeneRuler 100 bp Plus DNA ladder (ThermoFisher Scientific, USA)

One of the ubiquitous challenges of plant virus-based expression and VIGS vectors is instability of the heterologous sequence and loss of insertion sequences over time during replication cycles of the virus. Stability of the heterologous sequence insertions in pWX27, pWX68, and pWX56 was assayed over three weeks after rub-inoculation of 10-day-old seedlings using VPI launched infected leaf materials of pWX6, pWX27, pWX68, and pWX56 stored in a -80 °C freezer. At each time point, the youngest fully emerged leaf from the top of each plant was assayed for virus and insert stability by RT-PCR analysis with primers flanking the insertion sites (Additional file 1: Table S1). Amplicons were scored as full length insert band only **a**; full length insert plus smaller bands detected **b**; multiple bands with no full length insert detected **c**; single band of less than full-length insert, with size usually near that of wild type virus amplicon **d**; and no band **e** (Table 3, Fig. 5c; Additional file 2: Fig. S2; raw images in Additional file 3). pWX27 showed rapid loss of insertion sequence, with no samples showing full-length insert only by 14 dpi. pWX27 amplicons detected shifted from predominantly full-length or a mixture of amplicons with full-length insert to loss of most full length insert detection by 21 dpi (Table 3). In contrast, pWX68 retained some full-length only insert by 14 and 21 dpi. Even less full-length only insertion loss was observed for pWX56 VIGS and GFP insertion sequences (Table 3). Statistical comparison of the single band categories **a** (full-length insert single band) and **d** (single smaller than full-length band/near wild-type reversion) across insertion sites for the various constructs supported significantly improved insert stability of both pWX56 insertions compared to pWX27 and pWX68 insertions (Fig. 5a, b; see Additional file 1: Tables S2-S4 for *P*-values), indicating that dual insertion stabilizes the construct. Both GFP and triple VIGS insert stability in pWX56 were also examined through series of five passages to 10 plants by rub inoculation. GFP expression and VIGS photobleaching were detected in all five passages but were weak by the fifth passage (Additional file 1: Table S5; Additional file 2: Fig. S1).

**Table 3:** GFP and VIGS insert stability in constructs pWX27, pWX68, and pWX56

Construct	Insert	Rep.^**a**^	Time (dpi)	Total plants	a^**b**^	b	c	d	e
pWX27	GFP	1	7	20	9	10	0	0	1
		2	7	20	6	13	1	0	0
		3	7	20	11	8	0	1	0
		1	14	20	0	18	2	0	0
		2	14	20	0	13	4	2	1
		3	14	20	0	16	4	0	0
		1	21	20	0	5	12	2	1
		2	21	20	0	3	9	8	0
		3	21	20	0	3	12	5	0
pWX68	GFP	1	7	20	14	4	2	0	0
		2	7	20	2	11	4	3	0
		3	7	20	3	10	5	2	0
		1	14	20	10	6	2	2	0
		2	14	20	3	6	5	5	1
		3	14	20	2	4	6	8	0
		1	21	20	3	9	4	3	1
		2	21	20	0	0	12	7	1
		3	21	20	0	2	7	9	2
pWX56	GFP	1	7	20	7	6	1	4	2
		2	7	20	11	4	0	3	2
		3	7	20	8	5	1	1	5
		1	14	20	9	7	2	2	0
		2	14	20	3	14	1	2	0
		3	14	20	8	7	3	0	2
		1	21	20	2	8	2	4	4
		2	21	20	5	10	0	4	1
		3	21	20	0	12	2	5	1
pWX56	VIGS	1	7	20	6	9	5	0	0
		2	7	20	17	0	0	3	0
		3	7	20	0	16	0	0	4
		1	14	20	13	2	3	0	2
		2	14	20	6	12	0	1	1
		3	14	20	11	4	0	5	0
		1	21	20	12	3	0	1	4
		2	21	20	12	3	0	1	4
		3	21	20	4	4	1	1	10^c^

^**a**^Three experimental replicates (20 plants per construct in each), were tested by RT-PCR. Wild type pWX6 and healthy plants were used as positive and negative controls

^**b**^RT-PCR results per sample were scored into 5 different categories (**a**,**b**,**c**,**d** and **e**), based on band(s) observed: ***a*** full length insertion single band observed, ***b*** multiple bands observed; including expected full-length insertion and smaller bands indicating insert loss, ***c*** multiple bands indicating insert loss, with none representing full length insertion; ***d*** single band detected at less than full-length insertion size, usually close to size of wild type virus amplicon; ***e*** no bands detected with primer pair

^**c**^This replicate was excluded from statistical analyses since no amplification was observed in half of the samples

### Aphid transmission of pWX56

**Fig. 6:**
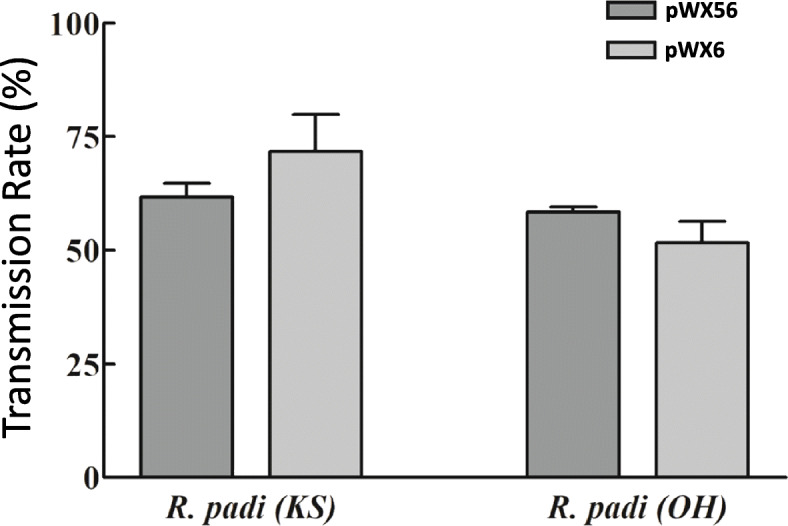
Aphid transmission of virus launched from pWX56 vs. wild type control pWX6. A total of 20 plants each for pWX6 and pWX56 were aphid inoculated with the respective constructs, and infectivity was scored by observation of mosaic symptoms and RT-PCR analysis (data not shown). No statistical significance was observed between pWX6 and pWX56 transmission or between transmission by Kansas and Ohio-derived *R. padi* colonies. Samples were taken from the newest fully emerged leaf of 17 dpi of aphid feeding plants for RT-PCR analysis using primers WX358/WX367 (1115 nt) for GFP insertion, and primers WX317/WX315 (965 nt) for VIGS insertion

Aphids (*Rhopalosiphum padi*) from both Ohio- and Kansas- originating colonies were used to test transmission of pWX56. Aphid transmission was successful as indicated by typical mosaic symptoms for both the wild type pWX6 cloned virus and pWX56, with no statistically significant difference in transmission rates for each construct or for *R. padi* colonies from Kansas versus Ohio. (Fig. 6). In pWX56 aphid-inoculated plants, photobleaching was observed (Additional file 2: Fig. S3A) and GFP expression was visualized by fluorescence microscopy (data not shown). VIGS and GFP insertions were further confirmed by RT-PCR detection of target genes (Additional file 2: Fig. S3B). VIGS insert sequence remained intact at 17 days post aphid inoculation, while GFP insert sequence showed a mix of intact and partially intact sequences at 17 days post aphid inoculation. Each of the two *R. padi* populations transmitted pWX6 and pWX56 at similar rates of 45–90% (9–18 infected plants per 20 plants infested by aphids with no statistically significant difference (*P* > 0.05) (Fig. 6).

## Discussion

We have demonstrated that MDMV can be used for simultaneous gene expression and multi-gene silencing in maize, the first pairing of both of these capabilities in a single virus-based tool for maize. This is significant as more than one gene could be evaluated simultaneously, with opposite overexpression and knockdown expression if desired, for functional analyses. This opens up the possibility of modifying other agriculturally important potyviruses/crops for gene expression and function studies. Unexpectedly, dual insertion also increased the stability of heterologous sequences in the virus compared to single site insertion constructs. As yet, the mechanism underlying the stabilization effect for dual insertion in this construct is not known. Similarly, since this is one of only two reported potyviruses that have been developed for VIGS, whether the ability of MDMV to be modified for successful multi-target VIGS and concurrent gene expression is commonplace but simply untested for potyviruses, or is a unique result of some rare feature of this virus remains unknown. Nevertheless, insertion sequences can be cloned and efficiently expressed simultaneously in pWX56, and constructs can be launched by transcript VPI. VPI has an estimated tenfold greater efficiency than initial launching of transcript RNA by rub-inoculation to plant leaves [59]. Once the construct has infected plants, early collections of leaves can be used for simple rub-inoculation to new maize plants for easy scale-up. Stable VIGS or GFP insertion and expression in MDMV demonstrate that MDMV could be used as a powerful tool for gene function analysis and gene expression in maize. Although all proof of concept work reported here was performed in maize genotypes Early Sunglow and Silver Queen, the pWX56 construct also infected and induced VIGS photobleaching in maize genotypes B73 and Mo17, suggesting that it can be utilized similarly in susceptible maize genotypes (preliminary data not shown). We also note for the benefit of end-users that optimal performance of pWX56 as reported here relies on optimal plant growth conditions: plants visibly stressed by poor growing conditions, in our case due to growth chamber malfunctions, showed marked and rapid loss of pWX56 insertion sequences when inoculated (results not shown).

Compared to other virus-based tools that are currently available for maize, (based on BMV, CMV, FoMV, SCMV, WSMV, BSMV, BYSMV, and MRFV), the multi-target and multi-function simultaneous VIGS/gene expression capability we report is a unique new development for grasses. Since each virus has unique features, and direct experimental and quantitative comparison of performance parameters of the various available virus-based tools for gene expression or gene silencing in maize have not been conducted, direct comparison of virus-based tools in maize is limited. However, published literature can aid in informing selection of a suitable tool for end-users based on different features such as carrying capacity, delivery method, insert stability, phenotype penetrance, and function, with the caveat that direct comparisons are imperfect due to the wide range of parameters utilized and measured in each system. As an additional caveat, it should also be noted that, in all virus systems examined and used for comparison, insertion stability as well as carrying capacity shows at least some sequence-dependence, thus there is not a strict carrying capacity or degree of stability for all possible insertion sequences.

The maximum sequence carrying capacity of constructs designed in the pattern of pWX56 was not examined. However, the total heterologous sequence carried by pWX56 (ca. 1.4 kb) is much greater than that reported for maize-infecting tools based on viruses with spherical virions, which are expected to have physical constraints on insertion size and carry silencing target sequences ca. 300 nt or smaller (BMV [20, 21], CMV [13], MRFV [22]). pWX56 sequence cargo size is comparable or slightly smaller than those reported for other viruses that also have rod-shaped virions, which may increase capsid length corresponding with increased sequence insertion. However, the modified rod-shaped viruses still have insertion size limitations, and generally have reduced performance and stability in maize with larger insertion sequences such as β-glucuronidase (GUS) (SCMV [27], WSMV [24–26], BSMV [31], and FoMV [29]). The BYSMV rhabdovirus tool is in a class by itself for overall carrying capacity, with one of the reported constructs carrying the large Cas9 gene as well as sequence encoding scaffolded guide RNA and a red fluorescent protein gene, ca. 5 kb in total [33]. However, it should be noted that launching these negative sense RNA virus-based constructs in maize is difficult, requiring transmission from planthoppers injected with crude sap from *Agrobacterium*-inoculated *Nicotiana benthamiana* [33].

Insertion stability in virus-based tools, for maize as well as other plants, is a common challenge, and can limit utility to only a few leaves above inoculation points [20, 23]. For insertion stability, pWX56 ranks among the most insert-stable virus-based tools for maize, such as the single protein expression tool based on the related virus SCMV [27], with insert retention and associated phenotypes observed systemically up to the last observation time points and topmost leaves, and up to three passages. However, its relative stability with progressive insert loss is not as impressive as the nearly complete insert retention over 60 days of the single-gene silencing tool based on MRFV [22]. Silencing efficacy of each of the target genes for pWX56, with gene expressions reduced to 39–46% of control expression levels, is within ranges expected for single-target VIGS BMV constructs (ca. 30–60% of control expression) [21], but has lower apparent silencing penetrance than some single-target VIGS constructs (13–33% of control expression in silenced tissue for BSMV [23], and 16–33% of control expression for MRFV [22])--noting again that differences in methodology and conditions make such comparisons imprecise. Protein expression of GFP from pWX56 is also visually robust, but as no quantitative assessments were performed here beyond insertion and expression stability, we cannot compare protein expression quantity among available reported tools. The qualitative properties of simultaneous opposite functions of gene silencing and gene expression, and the capability for multiple silencing targets, make pWX56 and derivatives of this design a novel addition to the maize virus-based toolbox.

## Conclusions

We demonstrated that an MDMV-derived vector can be used to express GFP protein and simultaneously silence three maize genes at the same time. We also showed that dual insertion enhanced the stability of inserted sequences.

## Materials and methods

### Plant materials and inoculation

MDMV OH5 was obtained from a Johnsongrass plant with mosaic symptoms collected from a field in Chillicothe, Ohio in October 2017 (public property collections and private property collections by permission of individual landowners were made, samples were not distinguished to original land ownership), and maintained in a greenhouse at Ohio State University (OSU) Wooster campus under OSU Institutional Biosafety Committee regulations. Plants were destructively sampled and not formally identified by taxonomic experts. Virus was transmitted from the source plant via rub inoculation by grinding leaf tissue in five volumes of 10 mM potassium phosphate, pH 7. Extract was rubbed onto leaves of 10 to 11 day old ‘Oh28’, ‘Early Sunglow’, and ‘Silver Queen’ between thumb and forefinger with inoculum mixed with 600-mesh silicon carbide (carborundum). All plant materials infected with modified virus constructs were grown in controlled growth chamber at 25 °C, 16 h light and 8 h dark conditions with light intensity of 13,000 lm.

### Sequencing MDMV OH5

Total RNA was isolated from four Johnsongrass samples (named MDMV OH3 to MDMV OH6) using Directzol RNA Miniprep Kit (Zymo Research, USA). Complementary DNA (cDNA) was synthesized and used for reverse transcription-PCR (RT-PCR). Primers specific to MDMV OH1 (MDMV-7065F, MDMV-4272F, MDMV genR1) were used for MDMV genome sequence amplification (Additional file 1: Table S1). Amplified DNA fragments were sequenced using the same primers above and MDMV-6241R. MDMV OH5 was used for subsequent studies.

Terminal sequences of MDMV were determined by 5′- and 3′-RACE. For 5′-RACE, 1 μg total nucleic acid extracted from MDMV-infected plants were first annealed with primer WX24 (Additional file 1: Table S1), then used for first strand cDNA synthesis using Superscript III reverse transcriptase (ThermoFisher Scientific, USA), followed by RNaseH treatment as described by the manufacturer (ThermoFisher Scientific, USA). The cDNA was then passed through a Monarch® PCR and DNA cleanup column (New England Biolabs, USA), quantified and G-tailed with 0.25 mM dGTP by terminal transferase (New England Biolabs, USA). The G-tailed cDNA was used as template for PCR with primers WX25 and WX27, followed by a second amplification PCR with primers WX26 and WX29 (Additional file 1: Table S1). PCR amplified DNA was either sequenced directly using primer WX27 or cloned into pMINIT 2.0 vector (New England Biolabs, USA), then subject to sequencing. For 3′-RACE, 1 μg total nucleic acid extracted from MDMV-infected plants were first annealed with primer WX292 (Additional file 1: Table S1), then used for first strand cDNA synthesis using Superscript III reverse transcriptase (ThermoFisher Scientific, USA), followed by RNaseH treatment as described by the manufacturer (ThermoFisher Scientific, USA). The cDNA was then passed through a Monarch® PCR and DNA cleanup column (New England Biolabs, USA). The cDNA was used as template for PCR with primers WX236 and WX293, followed by a second PCR with primers WX29 and WX237. Amplified DNA was either sequenced directly using primer WX27 or cloned into pMINIT 2.0 vector (New England Biolabs, USA) and then subject to sequencing.

### Creation of infectious cDNA clone of MDMV OH5

Full-length cDNA from MDMV OH5 was cloned into a binary vector pJL89 [57]. cDNA was prepared from 1 μg total RNA extracted from MDMV-infected plants, with first strand cDNA synthesis carried out using Superscript III reverse transcriptase and oligo d(T) primer, followed by RNaseH treatment as described by the manufacturer (ThermoFisher Scientific, USA). Full-length MDMV genomic sequence was amplified from the MDMV first strand cDNA using primers LRS764 and LRS765 (Additional file 1: Table S1). The amplified cDNA sequence was then cloned into SmaI and StuI-digested binary vector pJL89 using NEBuilder® HiFi DNA Assembly Master Mix (New England Biolabs, USA) as described by the manufacturer. Several infectious MDMV OH5 clones in pJL89 were obtained, one of which, named pWX6, was selected for sequencing and further analysis.

### Insertion of GFP at the N-terminal region of CP (pWX27)

Green fluorescent protein (GFP) gene sequence [60] was inserted using NEBuilder HiFi Assembly Master Mix (New England Biolabs Inc., USA) in-frame between pWX6 NIb and CP coding sequences with a duplicated cleavage sites inserted between nt 8386/8387: 15 nt (5′-CagGCcGGcGAgacc-3′, lower case indicating nucleotides changed to alter codons while retaining translated sequence) encoding amino acid sequence Q/AGET, cleaved by NIa-Pro at the beginning of the GFP gene; and 24 nt (5′-GAgGTtATcGAcGTgAAgCAcCAA-3′) encoding NIb cleavage site amino acid sequence of EVIDVKHQ/) at the end of GFP. Primers WX123/WX124 and WX126/WX127 were used to amplify the full-length GFP sequence, primers WX125/LRS764 and WX128/LRS765 for MDMV OH5 sequence, and LRS766/LRS769 were used to amplify the pJL89 vector (Additional file 1: Table S1). The GFP-encoding gene fragment was assembled into pWX6 using NEBuilder® HiFi DNA Assembly Master Mix (New England Biolabs, USA), and subsequent clones were tested for infectivity.

### Insertion of GFP at the N-terminal region of HCPro (pWX68)

GFP sequence was also cloned using NEBuilder HiFi Assembly Master Mix (New England Biolabs Inc., USA) in-frame between MDMV OH5 P1 and HCPro coding sequences with inserted NIb cleavage site sequence in pWX6 (nt 838/839), adding 12 nt 5′ (5′-GCcGAtCCtacc-3′), encoding amino acid sequence ADPT 5′, and 33 nt 3′ (5′- GAgGTAATcGAcGTgAAgCAcCAAGCcGGcGag-3′), encoding amino acid sequence EVIDVKHQ/AGE, cleaved by NIa-Pro) at the end of GFP. Nested primers WX36/WX37 and WX63/WX64 were used to amplify the full-length GFP gene sequence, and primers WX247 and WX250 were used to amplify pJL89 with MDMV sequence (Additional file 1: Table S1). The GFP gene fragment was assembled into pWX6 and recovered clones were tested for infectivity.

### Creation of a triple gene insertion in the N-terminal region of HCPro (pWX56)

Infectious clone pWX27 with GFP inserted between NIb and CP was used as backbone vector for a triple partial gene sequence cloning between P1 and HCPro. Three maize genes, magnesium chelatase (*Zm*ChlI, GenBank accession no. DQ084025, target region: 946–1193), lemon white 1 (*Zm*IspH, GenBank accession no. NM_001175829, target region: 740–988) and phytoene desaturase (*Zm*PDS, GenBank accession no. L39266, target region: 538–786) were selected for VIGS analysis. The triple gene fragment, 249 nt of each gene with total length of 747 nt, was synthesized (Eurofins Genomics, USA), cloned into pMINIT2.0 vector and verified by sequencing. The triple VIGS gene fragment was then amplified by PCR with primers WX251 and WX252, and the pWX27 backbone was amplified using primers WX247 and WX250, assembled using NEBuilder HiFi Assembly Master Mix (New England Biolabs Inc., USA) in-frame between P1 and HCPro to create pWX56. The triple VIGS DNA fragment contained nine additional 5′ nt (5′-GCcGAtCCt-3′) encoding amino acid sequence ADP at the beginning of the VIGS insertion, and 33 nt 3′ (5′- GAgGTAATcGAcGTgAAgCAcCAAGCcGGcGag-3′), encoding NIb cleavage site amino acid sequence of EVIDVKHQ/AGE, cleaved by NIa-Pro), between nt 838/839 of pWX27. Recovered clones were tested for infectivity and one infectious clone, pWX56, was selected for further analysis.

### Infectivity testing by vascular puncture inoculation of in vitro transcripts

All full-length virus constructs were amplified by polymerase chain reaction (PCR) from plasmid DNA templates using primers containing 5′ T7 promoter sequences, WX3 (Table S1) and reverse primer MDMV GenR1 [52] complementary to the 3′-most 17 nt of the MDMV 3-terminal sequence and adding a 21 nt of poly(A) to the virus-sense strand of the amplicon. PCR was performed using PrimeSTAR GXL DNA Polymerase from Takara Bio USA (Mountain View, CA) according to manufacturer’s instructions.

In vitro RNA transcripts were synthesized using T7 ARCA RNA transcription kit (New England Biolabs, USA) rather than the transcription kit used for previous work reporting MDMV OH1 infectious clone [52] and cleaned using with 2 M lithium chloride or the Monarch RNA cleanup kit (New England Biolabs, USA). Transcript quantity was estimated by NanoDrop (ThermoFisher Scientific, USA) and quality was assessed on non-denaturing 1% agarose 1X TBE (0.089 M Tris, 0.089 M boric acid, 0.002 M EDTA) gels.). Vascular puncture inoculation (VPI) was used to inoculate ‘Silver Queen’ maize seeds with 2.0 μg RNA transcript per seed as previously described [45, 50]. Inoculated seeds were germinated for two days at 30 °C, sown into sterilized soil, and grown in a growth chamber at 25 °C, 16 h light and 8 h dark conditions with light intensity of 13,000 lm. Infectivity of constructs was tested by reverse transcription polymerase chain reaction (RT-PCR) on leaves from individual plants with primers testing for MDMV (WX111 and WX112). RT-PCR was performed on fresh samples at each time point by grinding samples 1:20 (g/mL) in grape extraction buffer (GEB: 0.05 M sodium carbonate buffer pH 9.6, 2% polyvinylpyrrolidone-40, 0.2% bovine serum albumin, 0.05% Tween-20), then boiling the samples diluted 4 μl into 50 μl of at 95 °C for 10 min in GES buffer (0.1 M glycine-NaOH pH 9.0, 50 mM NaCl, 1 mM EDTA, 0.5% Triton X-100, and .01% beta-mercaptoethanol. One-step RT-PCR was performed with SuperScript III reverse transcriptase (Invitrogen, Carlsbad, CA) and GoTaq polymerase (Promega Corp., Madison, WI) at 52 °C for 40 min. Followed by 2 min at 94 °C and 32 cycles of 94 °C for 15 s, 55 °C for 20 s, and 72 °C for 1 min, ending with a 7 min. 72 °C extension.

### Rub inoculation scale-up from transcript-infected material

To scale up infection with constructs after VPI inoculation with transcripts, VPI-infected leaves were harvested as early as systemic symptoms could be robustly confirmed (7–10 dpi), to maximize virus harvest while minimizing replication cycles in which insert can be lost, and stored at − 80 °C for up to 3 months in 0.5 g aliquots. Frozen tissue was ground in five volumes of 10 mM pH 7 potassium phosphate buffer with 600-mesh silicon carbide (carborundum) added as abrasive. Extract was then rubbed onto leaves of 8 to 10-day old corn plants using thumb and forefinger, with 0.5 g frozen tissue providing enough inoculum for 20 plants and resulting in infection rates near 100%. Plants were symptomatic 5–7 days post rub inoculation.

### Visualization of GFP expression

GFP expression was visualized on leaves of *Z. mays* ‘Silver Queen’ at 7, and 21 days post rub inoculation. Images were taken with a Leica DFC460C (Leica Microsystems, USA) camera using fluorescence imaging with NIGHTSEA Green-only bandpass filter (NIGHTSEA, USA) at 3-s exposure to separate green fluorescence from maize autofluorescence. Brightfield images were taken at 1-s exposure.

### Western blotting

GFP protein expression was assessed by Western blotting. Plant tissue that tested positive by RT-PCR with primers WX358 and WX367 was saved at − 80 °C, for each time point of 7, 14, and 21 days post rub inoculation, and later used for Western blotting. Thawed tissue was ground in 1 ml of radioimmunoprecipitation assay (RIPA) buffer amended with one tablet cOmplete, Mini, EDTA-free Protease Inhibitor Cocktail (Millipore-Sigma, USA) and 300 μl 1 M dithiothreitol (DTT) per 10 ml of buffer. Ground tissue was centrifuged at 16,000 *g* at 4 °C for 20 min. Supernatant was transferred to new Eppendorf tubes and placed on ice for remainder of experiment. Total protein was determined using Pierce 660 nm Protein Assay kit and Pierce BCA Protein standards (ThermoFisher Scientific, USA) in a 96 well plate with 3 reps per sample.

Equal parts of leaf supernatant and 2X Laemmli sample buffer (Bio-Rad, USA) were mixed, boiled for 3 min and allowed to cool. Samples were loaded onto 4–20% Mini-PROTEAN TGX Stain-Free Protein Gels (Bio-Rad, USA) at 25 μg total protein per lane. Recombinant *E. coli* GFP Protein (Abcam, USA) was loaded at 5 ng as a positive control. Lysates were electrophoresed in Tris/Glycine/SDS buffer (25 mM Tris; pH 8.8, 200 mM glycine, 0.1% sodium dodecyl sulfate) at 200 V for 30 min. Proteins were transferred using Trans-Blot Turbo Transfer System on Trans-Blot Turbo Nitrocellulose Transfer Pack (Bio-Rad, USA) paper at 25 V for 7 mins. Membranes were blocked in 5% nonfat dry milk (NFDM) in TBS buffer (50 mM Tris and 150 mM NaCl, pH 7.5) for 1 h. Membranes were incubated on a shaker at room temperature for 1 h with 1:3000 Anti-GFP antibody (Abcam, USA) in 1% TBS-T (TBS buffer with 0.1% Tween-20). Membranes were washed for 10 min 3 times in TBS-T. Membranes were incubated with 1:2500 Goat Anti-Rabbit IgG H&L (Abcam, USA) for 1 h and then washed for 5 min 3 times in TBS-T. Proteins were visualized by chemiluminescence using Clarity Western ECL Substrate (Bio-Rad, USA) on a ChemiDoc XRS System (Bio-Rad, USA).

### RT-PCR insertion stability assays

Twenty plants were rub-inoculated with wild type and modified MDMV with GFP insert using verified VPI-sourced plant sap. Samples were collected at 7, 14, and 21 days post-rub inoculation from the youngest fully emerged leaf. Plant samples were screened by RT-PCR using the same method described above. Primers spanning whole GFP and GFP internal primers with MDMV OH5 either upstream or downstream GFP insertion were used for RT-PCR analysis. Amplified DNA was electrophoresed on 1% agarose gels. Leaf samples were collected as described above and analyzed for insertion stability and quantified using either semi-quantification RT-PCR.

Individual target genes of triple VIGS insertion were confirmed using one of MDMV OH5 specific primers either upstream (WX317) or downstream (WX315) of the triple VIGS insertion and specific primers (WX321 for *Zm*ChlI, WX325 for *Zm*IspH, and WX327 for ZmPDS) to each of three target genes of magnesium chelatase, lemon white1 and phytoene desaturase (*Zm*ChlI-IspH-PDS; triple VIGS). Photobleaching symptoms were observed and photographed, and chlorophyll content was measured with a MC-100 Chlorophyll Concentration Meter following manufacturer’s instructions (Apogee Instruments, USA). Three measurements were done for each leaf.

### RT-qPCR to quantify gene silencing

Leaf samples were collected 7, 14, and 21 days post-rub inoculation, total RNA was extracted as described above and quantified using either semi-quantitative RT-PCR or RT-qPCR. One microgram total RNA from each sample was used for cDNA synthesis in a 20 μl reaction by using iScript™ Reverse Transcription Supermix (Bio-Rad, USA). Primer efficiency was determined using 1 μl cDNA at 1/5, 1/10, 1/20, 1/40 and 1/160 dilutions (Additional file 1: Table S6). qPCR was carried out by using SsoAdvanced Universal SYBR Green Supermix (Bio-Rad, USA) in a Bio-Rad’s CFX96 real-time C1000 touch thermal cycler under conditions of 95 °C for 30 s, 39 cycles of: 95 °C for 10 s and 60 °C for 30 s, then 95 °C for 10 s, melt curve 65 °C to 95 °C with an increment of 0.5 °C, 5 s. All samples were run with cDNA dilution of 1:5, cDNA derived from 10 ng total RNA. Gene quantification and analysis was done on target genes of *Zm*ChlI, *Zm*IspH, *Zm*PDS as well as GFP, MDMV, and along with reference genes of membrane protein PB1A10.07c (MEP) and folypolyglutamate synthase (FPGS) [61]. Primers used for RT-qPCR analysis are shown in (Additional file 1: Table S1, S6). Ct was determined using E = 10^[− 1/slope] (Additional file 1: Table S6).

### Aphid transmission assays

Wild type MDMV OH5 and modified MDMV OH5 derived VIGS were transmitted by *Rhopalosiphum padi*. *R. padi* were maintained on virus-free ‘Early Sunglow’ maize plants in the cages at 25 °C with a photoperiod of 15 h light/ 9 h dark. ‘Silver Queen’ maize plants were inoculated by VPI with in vitro RNA transcript. The infected tissues were collected and kept at − 80 °C, which were used for rub inoculation. The virus source plants were prepared by rub inoculating seven-day-old seedlings ‘Early Sunglow’ with the VPI tissue and the plants were kept at 20 °C with a photoperiod of 15 h light/ 9 h dark for 14 days. The leaves with strong MDMV symptoms (usually the 1/3 from tip in the 2nd leaf of plant) were sprayed with 10% sucrose, and were left to dry out before collection. The collected leaf was then cut into smaller pieces (around 1 cm^2^) and were placed in a small box (around 10 cm^3^) lined with wet tissue paper for maintaining the moisture. *R. padi* were then fed on source plants in the box for 10 min (acquisition access period), and after acquisition period, 10 aphids were moved onto the corn whorl per plant with 20 healthy ‘Early Sunglow’ plants in total for each treatment. The plants were covered with a plastic tube for 3 days, and then aphids were removed by NUVAN PROSTRIPS (AMVAC Chemical Corporation, USA) for 2–4 h. Symptoms was scored at 7, and 14 days post-inoculation and infection was further confirmed by RT-PCR using primers WX317/WX315 for VIGS, and WX368/WX357 for GFP insertion.

### Statistical analysis

Insert stability in MDMV OH5 constructs at 7, 14, and 21 dpi was analyzed statistically. Constructs of pWX27, pWX68, and pWX56, three replicates of 20 plants inoculated per construct, were examined for integrity of insertion sequences using RT-PCR with primers flanking the insertion sites. Linear mixed model was used to analyze the effects of constructs, time, and their interactions on band types. F-statistics and probability values from the fit of linear mixed models to arcsine-square root transformed bands data. Data were arcsine-square-root transformed prior to analysis to stabilize variance. Since data were collected as temporal repeated measures on the same experimental units and as such were correlated in time, the random _residual_ statement and type option in GLIMMIX were used to account for, and model, the covariance structure (compound symmetry) of the within-subject data. Models were fitted using the GLIMMIX procedure of SAS.

## Supplementary Information

**Additional file 1: Table S1**. Primers used in cloning, vector construction, RT-PCR and RT-qPCR. **Table S2**. Summary statistics from linear mixed model analyses of the effects of constructs, time, and their interactions on band types. **Table S3**. Probability values (*p*-values) for pairwise comparisons of least squares means between constructs at fixed levels of assessment time from linear model mixed analyses of the effects of constructs, and assessment time on arcsine-square root-transformed bands data. **Table S4**. Probability values (p-values) for pairwise comparisons of least squares means between assessment times at fixed construct from linear model mixed analyses of the effects of constructs, and assessment time on arcsine-square root-transformed bands data. **Table S5**. Passaging test of pWX56 for GFP expression and VIGS photobleaching. **Table S6**. Target and reference genes used in RT-qPCR analysis.

**Additional file 2: Figure S1**. Analysis of pWX56 infected plants. A. RT-PCR analysis of target gene insertion of pWX56 plant 40 days post VPI, lanes: MDMV-VIGS (primers WX317/WX176: 938 bp); MDMV-*Zm*ChlI (primers WX291/WX321: 382 bp); MDMV-*Zm*IspH (primers WX291/WX325:636 bp), MDMV-*Zm*PDS (primers WX327/WX315:413 bp); pWX56-infected plant (lanes 2–5); pWX6-infected plant (lane 6, with primers WX317/WX315: 176 bp); pWX56 DNA control (primers WX317/WX315: 965 bp) (lane 8). B. Chlorophyll content measurement (μmol per m^2^) of newest fully emerged leaf of each plant: healthy (HC), pWX6, and pWX56. C. Representative images of GFP and photobleaching after pWX56 rub-inoculation passages (see **Table S5**). 0P = plant rub-inoculated from VPI tissue, 1P-5P = plants rub-inoculated with 14 dpi pooled tissue from prior inoculation, all shown 14 dpi. Images were taken with a Leica DFC460C camera using fluorescence imaging with NIGHTSEA Green-only bandpass filter at 3-s exposure and bottom panel images are taken with the same camera without fluorescence at 1 s exposure. D. pWX56-infected whole plant silencing 90 days post inoculation. **Figure S2**. Representative gels showing GFP and VIGS insertion stability analysis by RT-PCR. A. pWX27-inoculated plants tested with primers WX111/112 (Table S1) spanning NIb/CP insertion site. B. pWX68-inoculated plants tested with primers WX315/317 (**Table S1**) spanning P1/HCPro insertion site. C. pWX56 [GFP] tested with primers WX358/367 (**Table S1**) spanning NIb/CP insertion site. D. pWX56 [VIGS] tested with primers WX315/317 (**Table S1**) spanning P1/HCPro insertion site. For each construct, assays from 20 rub-inoculated plants are shown in first lanes, followed by control assays from either five pWX6-inoculated control plants and five mock-inoculated control plants (A-B) or five pWX27-inoculated, five pWX6-inoculated, and five mock-inoculated control plants (C-D). Samples of the newest fully emerged leaf from top of each test plant was collected and fresh tissue used for one-step RT-PCR at 7, 14, and 21 days post inoculation as indicated, with gradient of bands shown spliced from three gels in each rightmost panel. Expected full-length amplicon sizes for test construct (top) vs. pWX6 no-insert control (bottom) with arrows to the left of each leftmost panel. **Figure S3**. Analysis of pWX56 infected plants transmitted by aphids *R. padi*. A. Photobleaching phenotype of pWX56 infected plants cv. Early Sunglow by aphid *R. padi* 28 days post aphid transmission. HC: healthy plant; WT: wild type pWX6 infected plant; L1-L5 leaves of pWX56 infected plant from newest (L1) to oldest (L5) leaves, photographed at 28 days post transmission. B. Aphid transmission of pWX56 inoculum using the newest fully emerged young leaves from top of the plants were collected, GFP and VIGS were assayed at 17 days post inoculation by PCR with primers spanning the insertion site. pWX6: wild type MDMV OH5 transmitted plants; controls: pWX56 plasmid DNA and PCR master mix without template. Expected amplified PCR fragment sizes: pWX56 GFP full-length insert vs WT: WX358/WX367 (1115 bp vs. 335 bp); pWX56 VIGS WX317/WX315 (965 bp vs. 176 bp).

**Additional file 3: **Raw gel and blot images for Fig. 3. Raw gel images for Additional file 2: **Figure S2**.

## Data Availability

Reasonable requests will be fulfilled provided that written materials transfer and/or licensing agreements, and appropriate APHIS permits and containment requirements are in place. Requests should be directed to the corresponding author. The complete sequence of the cloned Johnsongrass-derived field MDMV isolate was named MDMV OH5 and deposited as GenBank accession no. MN615724.

## Abbreviations

CP: Coat protein
cDNA: Complementary DNA
GFP: Green fluorescent protein
HCPro: Helper component proteinase
MDMV: Maize dwarf mosaic virus
NIb-RdRP: Nuclear inclusion b-RNA-dependent RNA polymerase
*R. padi*: *Rhopalosiphum padi*
RT-PCR: Reverse transcription-polymerase chain reaction
*Zm*ChlI: *Zea mays* magnesium chelatase
*Zm*IspH: *Zea mays* lemon white1
*Zm*PDS: *Zea mays* phytoene desaturase (*Zm*ChlI-IspH-PDS
VIGS: Virus-induced gene silencing
VPI: Vascular puncture inoculation
