# Supplementary material for: Simultaneous gene expression and multi-gene silencing in Zea mays using maize dwarf mosaic virus
Source: BMC Plant Biol. 2021 May 5;21:208. doi: 10.1186/s12870-021-02971-1 (PMC8097858; doi:10.1186/s12870-021-02971-1)
Supplement: Supplementary file 1 — Additional file 1: Table S1. Primers used in cloning, vector construction, RT-PCR and RT-qPCR. Table S2. Summary statistics from linear mixed model analyses of the effects of constructs, time, and their interactions on band types. Table S3. Probability values (p-values) for pairwise comparisons of least squares means between constructs at fixed levels of assessment time from linear model mixed analyses of the effects of constructs, and assessment time on arcsine-square root-transformed bands data. Table S4. Probability values (p-values) for pairwise comparisons of least squares means between assessment times at fixed construct from linear model mixed analyses of the effects of constructs, and assessment time on arcsine-square root-transformed bands data. Table S5. Passaging test of pWX56 for GFP expression and VIGS photobleaching. Table S6. Target and reference genes used in RT-qPCR analysis. [file 12870_2021_2971_MOESM1_ESM.docx]

Table S1. Primers used in cloning, vector construction, RT-PCR and RT-qPCR.

| Primer name | Sequence (5'-3') | Description |
| --- | --- | --- |
| LRS752 | ATCTCGTTGGGGATGTCTTG | Forward FPGS, GenBank NM_001350861.1, nt 1634-1653 |
| LRS753 | AGCACCGTTCAAATGTCTCC | Reverse FPGS, GenBank NM_001350861.1, nt 1746-1765 |
| LRS756 | TGTACTCGGCAATGCTCTTG | Forward MEP, GenBank NM_001137018.1, nt 1195-1214 |
| LRS757 | TTTGATGCTCCAGGCTTACC | Reverse MEP RT-qPCR, GenBank NM_001137018.1, nt 1378-1397 |
| LRS764 | GTTCATTTCATTTGGAGAGGGAAAACAACAAGACTCAAC | Forward primer for assembly of MDMV OH5 into pJL89 |
| LRS765 | GGAGATGCCATGCCGACCCTTTTTTTTTTTTTTTTGTCTCTCAC | Forward primer for assembly of MDMV OH5 into pJL89 |
| LRS766 | CCTCTCCAAATGAAATGAACTTCC | Forward pJL89, nt 4678-4701 |
| LRS769 | GGGTCGGCATGGCATCTC | Reverse pJL89, nt 27-44 |
| MDMV-4272F | TTTGAATGGACCGTGGACAGTC | Forward MDMV OH1, GenBank JQ403608.1, nt 7474-7495 |
| MDMV-6241R | TGTCTCTCACCACGAAACTCG | Reverse MDMV OH1, GenBank JQ403608.1, nt 9423-9443 |
| MDMV-7065F | CTGATTTGTTTGAGAGAGC | Forward MDMV OH1, GenBank JQ403608.1, nt7089-7107 |
| MDMV GenR1 | TTTTTTTTTTTTTTTTTTTTTGTCTCTCACCACGAAAC | From Stewart et al. (2012). Reverse MDMV OH1, GenBank JQ403608.1, nt 9426-9458. |
| WX3 | CGTACGCGTTAATACGACTCACTATAGAAAACAACAAGACTCAACACAACACAACC | Forward primer with T7 promoter sequence for MDMV RNA transcripts, T7 promoter sequence + MDMV OH5 nt 2-29 |
| WX24 | CGCCTCCTTCCAATGAACTC | Reverse MDMV OH1, GenBank JQ403608.1, nt 509-528 |
| WX25 | GGTTTGACAGGTTGCTGCTT | Reverse MDMV OH1, GenBank JQ403608.1, nt 419-438 |
| WX26 | CCGCCACTTCTTTCCACATC | Reverse MDMV OH1, GenBank JQ403608.1, nt 349-368 |
| WX27 | GGCCACGCGTCGACTAGTACCCCCCCCCCC | Forward primer for MDMV 5'-RACE cDNA tailing |
| WX29 | GGCCACGCGTCGACTAGTAC | Nested forward primer for MDMV 5'-RACE |
| WX36 | CGATCCTACCATGGGATCGATGCATCATCATCATCATCATGTGAGC | Forward primer for GFP amplification from pWX68, GGATCCTA+ nt 1-38 GFP sequence |
| WX37 | GCCTCGCCGGCTTGGTGCTTCACGTCGATTACCTCCTTGTACAGCTCGTCCATGCCGTGAGTG | Reverse primer for GFP amplification for pWX68, MDMV OH5 NIb +nt 719-746 GFP sequence |
| WX63 | TACACTACAAACATTCAAGAAATAGAACACTACGCCGATCCTACCATGGGATCGATGCATCATCATCATCATCATGTG | Forward primer for GFP amplification for pWX68, MDMV OH5 nt 806-828+GFP sequence |
| WX64 | TGAACCCCCTCCAAAAATCAGCTGCTTGTGGGTCTGCCTCGCCGGCTTGGTGCTTCACGT | Reverse primer for GFP amplification for pWX68, overlapping with part of WX37 primer |
| WX111 | CTGTACTGCTTCATACCACCTATC | Reverse MDMV OH5, nt 8768-8791 |
| WX112 | CTGGTGCTAAGGTTTCAGAAG | Forward MDMV OH5, nt 8286-8306 |
| WX123 | CAGGCCGGCGAGACCATGGGATCGATGCATCATCATC | Forward primer for GFP amplification for pWX27 |
| WX124 | GTGCTTCACGTCGATAACCTCCTTGTACAGCTCGTCCATGC | Reverse primer for GFP amplification for pWX27 |
| WX125 | ATGTTTGACATCTATTACTTCATCTGC | Reverse MDMV OH5, nt 8360-8386 |
| WX126 | TTAGCAGATGAAGTAATAGATGTCAAACATCAGGCCGGCGAGACCATGGGATCGA | Forward primer for GFP amplification for pWX27 |
| WX127 | CTGTCCGACATCAACATTTTCACCAGCTTGGTGCTTCACGTCGATAACCTCCTTGTACAGCTCG | Reverse primer for GFP amplification for pWX27 |
| WX128 | CAAGCTGGTGAAAATGTTGATGTCG | Forward MDMV OH5, nt 8387-8411 |
| WX176 | CGAGATTTATCGTAGGTGTGTGC | Reverse MDMV OH5, nt 922-944 |
| WX236 | CACAAATGACAGTTGTCATGAGTGG | Forward 3'-RACE PCR of MDMV OH5, nt 8814-8838 |
| WX237 | GAGAATGCATCTCCAACTTTC | Forward 3'-RACE PCR of MDMV OH5, nt 8945-8965 |
| WX247 | GCGTAGTGTTCAATTTCTTGAATGTTG | Reverse MDMV OH5, nt 813-840 |
| WX250 | AGACCCACAAGCAAATGATTTTTGG | Forward MDMV OH5, nt 841-865 |
| WX251 | TACACTACAAACATTCAAGAAATTGAACACTACGCCGATCCTAAGCTCCAGCAGCAGATATCATCTGCACG | Forward triple VIGS (ZmChII-IspH-PDS) |
| WX252 | TGTACCCCCTCCAAAAATCATTTGCTTGTGGGTCTGCCTCGCCGGCTTGGTGCTTCACGT | Reverse triple VIGS (ZmChII-IspH-PDS) |
| WX291 | CGTGGCAAAATGTTATGCGACAC | Forward MDMV OH5, nt 664-686 |
| WX292 | GGCCACGCGTCGACTAGTACTTTTTTTTTTTTTTTTT | Reverse MDMV primer for cDNA synthesis for 3'-RACE |
| WX293 | CTACTACTACTAGGCCACGCGTCGACTAGTAC | Reverse MDMV primer for PCR amplification for 3'-RACE |
| WX315 | ACAAGGCCATACGCTTGCCACAC | Reverse MDMV OH5, nt 949-971 |
| WX317 | CAGCTTGGATTACACTACAAAC | Forward MDMV OH5, nt 796-817 |
| WX321 | AGCAATGTCCTCCACGGTGAC | Reverse ZmChII, GenBank DQ084025.1, nt 1123-1143 |
| WX325 | GAGCTAGACCCATCAAGTTG | Reverse ZmIspH, GenBank NM_001175829.2, nt 923-942 |
| WX327 | CGAGCTTAGGATTGAGGATC | Forward ZmPDS, GenBank L39266.1, nt 540-559 |
| WX358 | GGTTTCAGAAGACGAACTAAACG | Forward MDMV OH5, nt 8296-8318 |
| WX367 | GTACTGACACTGAACCCGAAG | Reverse MDMV OH5, nt 8610-8630 |
| WX522 | TCGGGTTCAGTGTCAGTACC | Forward MDMV OH5, nt 8612-8631 |
| WX523 | TCAAATTCAGCCCTGGTTGC | Reverse MDMV OH5, nt 8750-8769 |
| WX526 | TCCTCATCGGCTCTGGTAAC | Forward ZmChII, GenBank DQ084025.1, nt 761-780 |
| WX527 | CCCTCTCCTCCACGATCTTC | Reverse ZmChII, GenBank DQ084025.1, nt 870-889 |
| WX528 | AAGGAGAACGGCAACCAGTA | Forward ZmIspH, GenBank NM_001175829.2, nt 401-420 |
| WX529 | GGGGTTGTGGATGATTTCGT | Reverse ZmIspH, GenBank NM_001175829.2, nt 546-565 |
| WX534 | GTCCCAAACTGTGAGCCTTG | Forward ZmPDS, GenBank, L39266.1, nt 1546-1565 |
| WX535 | TTCTGGCTCCTGAGTGTGAG | Reverse ZmPDS, GenBank L39266.1, nt 1705-1724 |
| WX540 | AAGGGCATCGACTTCAAGGA | Forward GFP, GenBank JN811690.1, nt 2266-2285 |
| WX541 | GGCGGATCTTGAAGTTCACC | Reverse GFP, GenBank JN811690.1, nt 2376-2395 |

Table S2. Summary statistics from linear mixed model analyses of the effects of constructs, time, and their interactions on band types.

| Factors* | Full length insert only | |  | Near wild-type reversion only | |
| --- | --- | --- | --- | --- | --- |
|  | F-values | P-values |  | F-values | P-values |
| Constructs | 5.74 | 0.0073 |  | 4.49 | 0.0181 |
| Time | 10.85 | 0.0011 |  | 10.23 | 0.0014 |
| Constructs x Time | 3.16 | 0.0306 |  | 3.37 | 0.0243 |

^*^Main and interaction effects of constructs (pWX27 GFP, pWX68 GFP, pWX56 GFP, pWX56 VIGS), and three times of assessment (7, 14, and 21 days).

Table S3. Probability values (p-values) for pairwise comparisons of least squares means between constructs at fixed levels of assessment time from linear model mixed analyses of the effects of constructs, and assessment time on arcsine-square root-transformed bands data.

| **Time** | **Contrasts** | **Full length insert only** | **Near wild-type reversion only** |
| --- | --- | --- | --- |
| 7 | pWX68 GFP vs pWX27 GFP | 0.426 | 0.145 |
|  | pWX56 GFP vs pWX27 GFP | 0.932 | 0.007 |
|  | pWX56 VIGS vs pWX27 GFP | 0.438 | 0.239 |
|  | pWX56 VIGS vs pWX56 GFP | 0.527 | 0.097 |
| 14 | pWX68 GFP vs pWX27 GFP | 0.013 | 0.002 |
|  | pWX56 GFP vs pWX27 GFP | 0.012 | 0.067 |
|  | pWX56 VIGS vs pWX27 GFP | 0.002 | 0.801 |
|  | pWX56 VIGS vs pWX56 GFP | 0.395 | 0.131 |
| 21 | pWX68 GFP vs pWX27 GFP | 0.471 | 0.474 |
|  | pWX56 GFP vs pWX27 GFP | 0.051 | 0.868 |
|  | pWX56 VIGS vs pWX27 GFP | 0.000 | 0.050 |
|  | pWX56 VIGS vs pWX56 GFP | 0.051 | 0.089 |

Table S4. Probability values (p-values) for pairwise comparisons of least squares means between assessment times at fixed construct from linear model mixed analyses of the effects of constructs, and assessment time on arcsine-square root-transformed bands data.

| Construct | Contrasts | Full length insert only | Near wild-type reversion only |
| --- | --- | --- | --- |
| pWX27GFP | 7 vs 14 days | 0.000 | 0.728 |
|  | 7 vs 21 days | 0.000 | 0.000 |
| pWX68GFP | 7 vs 14 days | 0.642 | 0.009 |
|  | 7 vs 21 days | 0.008 | 0.001 |
| pWX56GFP | 7 vs 14 days | 0.358 | 0.342 |
|  | 7 vs 21 days | 0.098 | 0.771 |
| pWX56VIGS | 7 vs 14 days | 0.517 | 0.450 |
|  | 7 vs 21 days | 0.957 | 0.814 |

Table S5. Passaging test of pWX56 for GFP expression and VIGS photobleaching

|  | **0P^a^** | **1P** | **2P** | **3P** | **4P** | **5P** |
| --- | --- | --- | --- | --- | --- | --- |
| **pWX6**  **Mosaic symptoms** | 10 | 10 | 10 | 9 | 8 | 6 |
| **pWX56**  **Mosaic symptoms** | 10 | 10 | 10 | 10 | 8 | 8 |
| **pWX56**  **GFP** | 10 | 10 | 10 | 8 | 8 | 6^b^ |
| **pWX56**  **VIGS** | 10 | 10 | 10 | 10 | 8 | 8^b^ |

^a^All numbers are out of 10 inoculated plants. Passage 0 (0P) was rub-inoculated with frozen VPI tissue. For each successive passage, pooled leaves from all plants in the previous passage were collected at 10 dpi and used to rub-inoculate 10 new plants. Plants were scored for symptoms, GFP, and photobleaching (VIGS phenotype) at 14 dpi.

^b^GFP and photobleaching were detected at the fifth passage but were very weak compared to earlier passages, see also Fig. S1C.

Table S6. Target and reference genes used in RT-qPCR analysis.

| **Gene name** | **Transcript ID** | **Gene product** | **Primer ID** | **Product size (bp)** | **PCR efficiency^a^** |
| --- | --- | --- | --- | --- | --- |
| FPGS | NM_001350861.1 | folypolyglutamate synthase | LRS752/753 | 132 | 2.04 |
| MEP | NM_001137018.1 | membrane protein PB1A10.07c | LRS756/757 | 203 | 2.11 |
| GFP | JN811690.1 | green fluorescent protein | WX540/541 | 130 | 1.81 |
| MDMV OH5 | MN615724 | maize dwarf mosaic virus | WX522/523 | 158 | 2.12 |
| *Zm*ChlI | DQ084025 | magnesium chelatase | WX526/527 | 129 | 2.08 |
| *Zm*IspH | NM_001175829 | *Zea mays* lemon white1 (lw1) | WX528/529 | 165 | 1.86 |
| *Zm*PDS | L39266 | *Zea mays* phytoene desaturase | WX534/535 | 179 | 1.91 |

^* q^PCR efficiency was determined from slope (E=10^[-1/slope]). Serial dilutions of cDNA synthesized from equivalent of total RNA of 10, 5, 2.5 and 1.25 ng were used for RT-qPCR for the calculation of slope for each target and reference gene.
