# Supplementary material for: Simultaneous gene expression and multi-gene silencing in Zea mays using maize dwarf mosaic virus
Source: BMC Plant Biol. 2021 May 5;21:208. doi: 10.1186/s12870-021-02971-1 (PMC8097858; doi:10.1186/s12870-021-02971-1)
Supplement: Supplementary file 2 — Additional file 2: Figure S1. Analysis of pWX56 infected plants. A. RT-PCR analysis of target gene insertion of pWX56 plant 40 days post VPI, lanes: MDMV-VIGS (primers WX317/WX176: 938 bp); MDMV-ZmChlI (primers WX291/WX321: 382 bp); MDMV-ZmIspH (primers WX291/WX325:636 bp), MDMV-ZmPDS (primers WX327/WX315:413 bp); pWX56-infected plant (lanes 2–5); pWX6-infected plant (lane 6, with primers WX317/WX315: 176 bp); pWX56 DNA control (primers WX317/WX315: 965 bp) (lane 8). B. Chlorophyll content measurement (μmol per m2) of newest fully emerged leaf of each plant: healthy (HC), pWX6, and pWX56. C. Representative images of GFP and photobleaching after pWX56 rub-inoculation passages (see Table S5). 0P = plant rub-inoculated from VPI tissue, 1P-5P = plants rub-inoculated with 14 dpi pooled tissue from prior inoculation, all shown 14 dpi. Images were taken with a Leica DFC460C camera using fluorescence imaging with NIGHTSEA Green-only bandpass filter at 3-s exposure and bottom panel images are taken with the same camera without fluorescence at 1 s exposure. D. pWX56-infected whole plant silencing 90 days post inoculation. Figure S2. Representative gels showing GFP and VIGS insertion stability analysis by RT-PCR. A. pWX27-inoculated plants tested with primers WX111/112 (Table S1) spanning NIb/CP insertion site. B. pWX68-inoculated plants tested with primers WX315/317 (Table S1) spanning P1/HCPro insertion site. C. pWX56 [GFP] tested with primers WX358/367 (Table S1) spanning NIb/CP insertion site. D. pWX56 [VIGS] tested with primers WX315/317 (Table S1) spanning P1/HCPro insertion site. For each construct, assays from 20 rub-inoculated plants are shown in first lanes, followed by control assays from either five pWX6-inoculated control plants and five mock-inoculated control plants (A-B) or five pWX27-inoculated, five pWX6-inoculated, and five mock-inoculated control plants (C-D). Samples of the newest fully emerged leaf from top of each test plant was collected and fresh tissu [file 12870_2021_2971_MOESM2_ESM.pptx]

## Slide 1
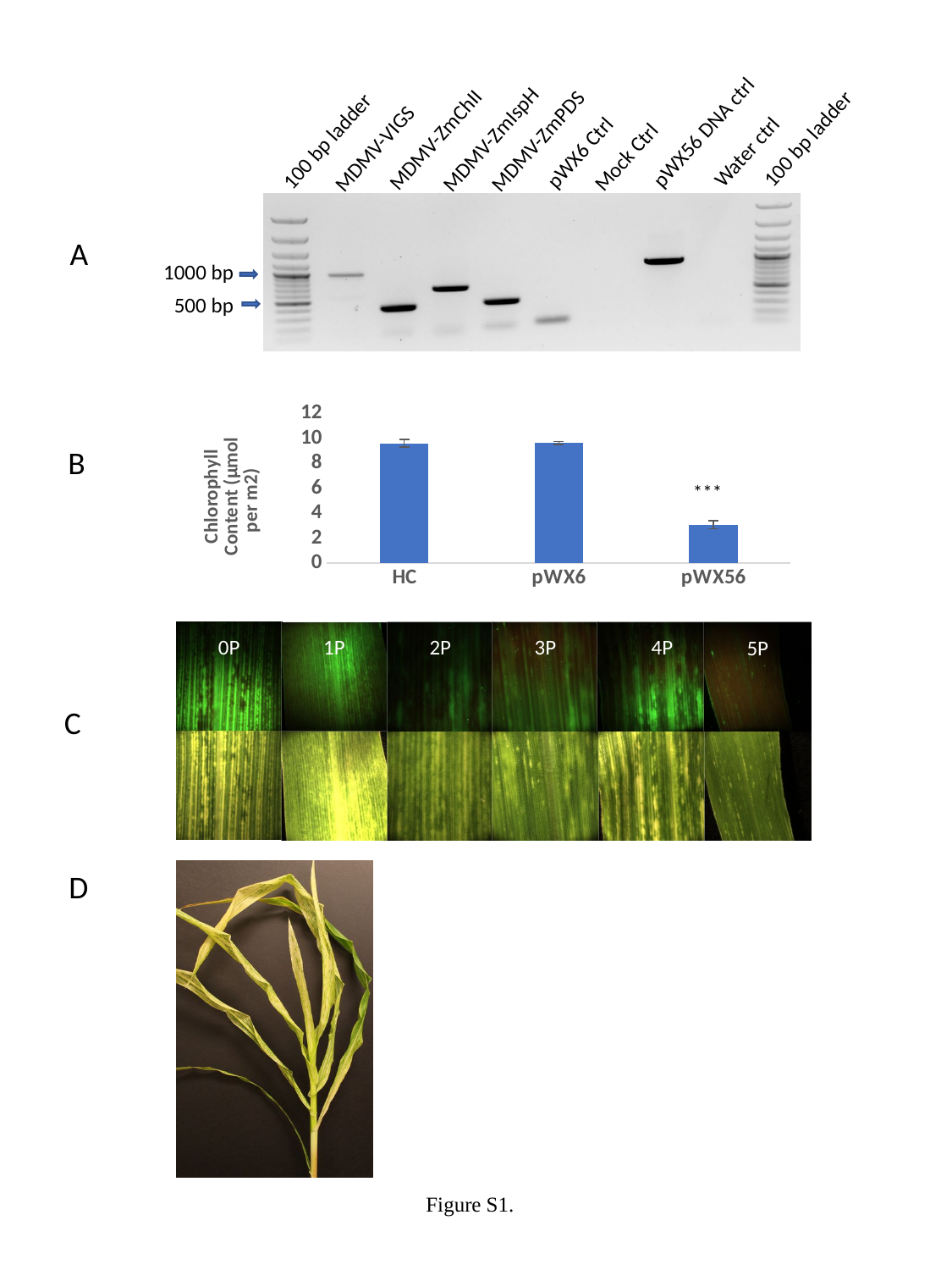

pWX56 DNA ctrl
MDMV-ZmChII
MDMV-ZmPDS
100 bp ladder
Water ctrl
MDMV-ZmIspH
pWX6 Ctrl
100 bp ladder
Mock Ctrl
MDMV-VIGS
A
1000 bp
500 bp
### Chart
| Category | mean |
|---|---|
| HC | 9.62 |
| pWX6 | 9.6475 |
| pWX56 | 3.0999999999999996 |B
***
5P
0P
1P
2P
3P
4P
C
Figure S1.
D

## Slide 2
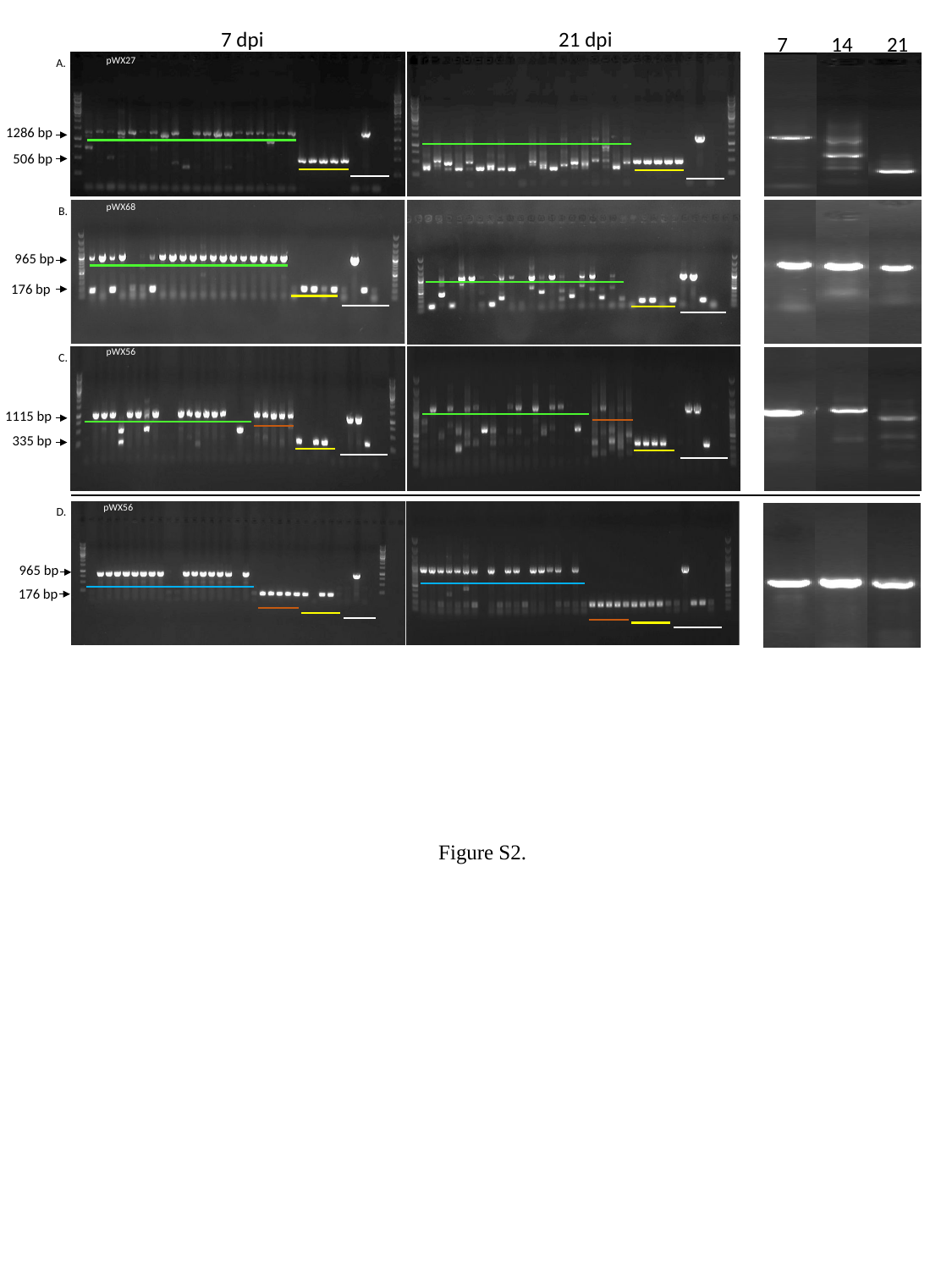

21 dpi
7 dpi
7 14 21
A.
pWX27
1286 bp
506 bp
B.
pWX68
965 bp
176 bp
C.
pWX56
1115 bp
335 bp
pWX56
D.
965 bp
176 bp
Figure S2.

## Slide 3
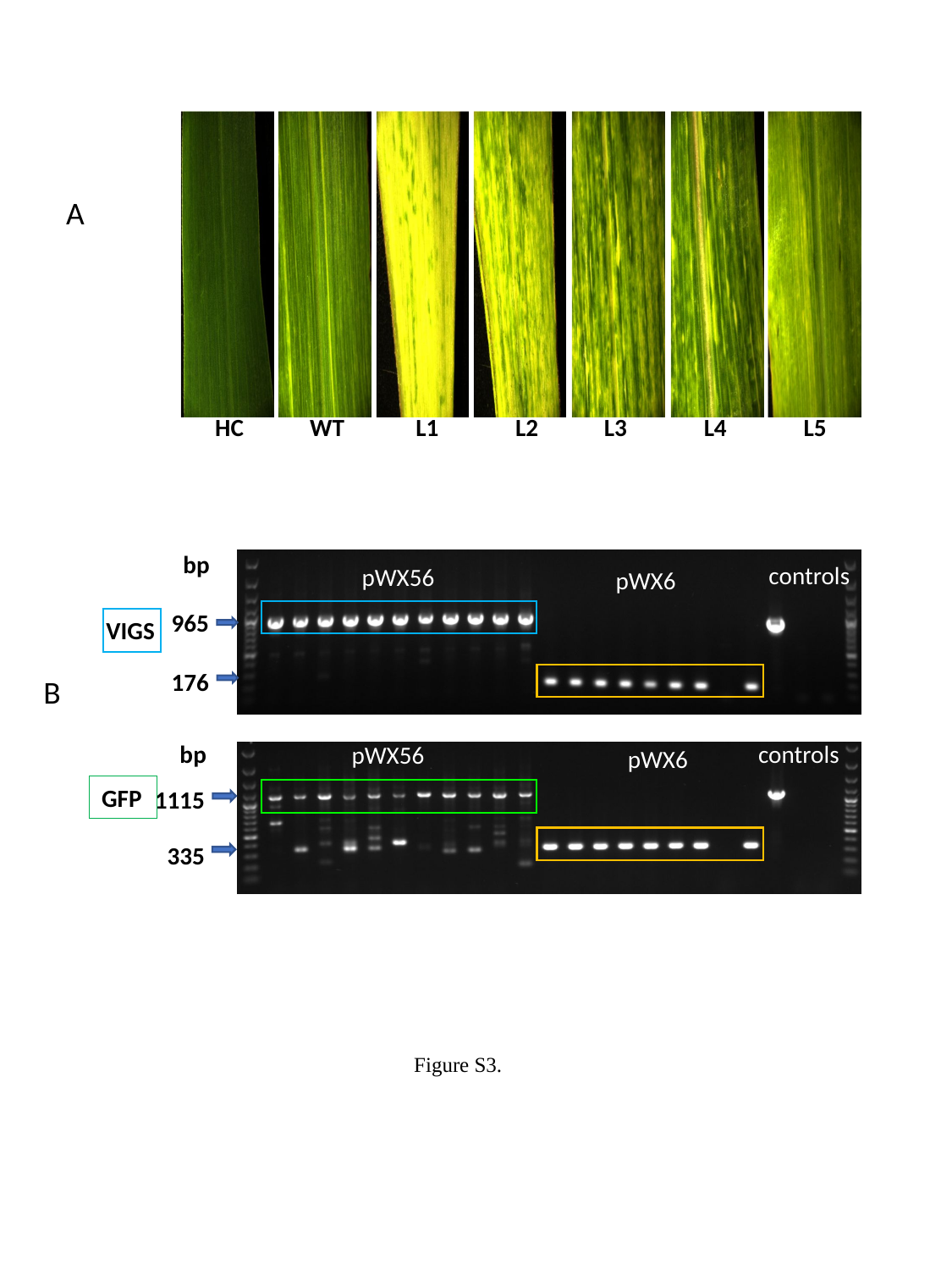

HC WT L1 L2 L3 L4 L5
A
bp
controls
pWX56
pWX6
965
VIGS
176
bp
controls
pWX56
pWX6
GFP
1115
335
B
Figure S3.
