# Supplementary material for: Simultaneous gene expression and multi-gene silencing in Zea mays using maize dwarf mosaic virus
Source: BMC Plant Biol. 2021 May 5;21:208. doi: 10.1186/s12870-021-02971-1 (PMC8097858; doi:10.1186/s12870-021-02971-1)
Supplement: Supplementary file 3 — Additional file 3: Raw gel and blot images for Fig. 3. Raw gel images for Additional file 2: Figure S2. [file 12870_2021_2971_MOESM3_ESM.pptx]

## Slide 1
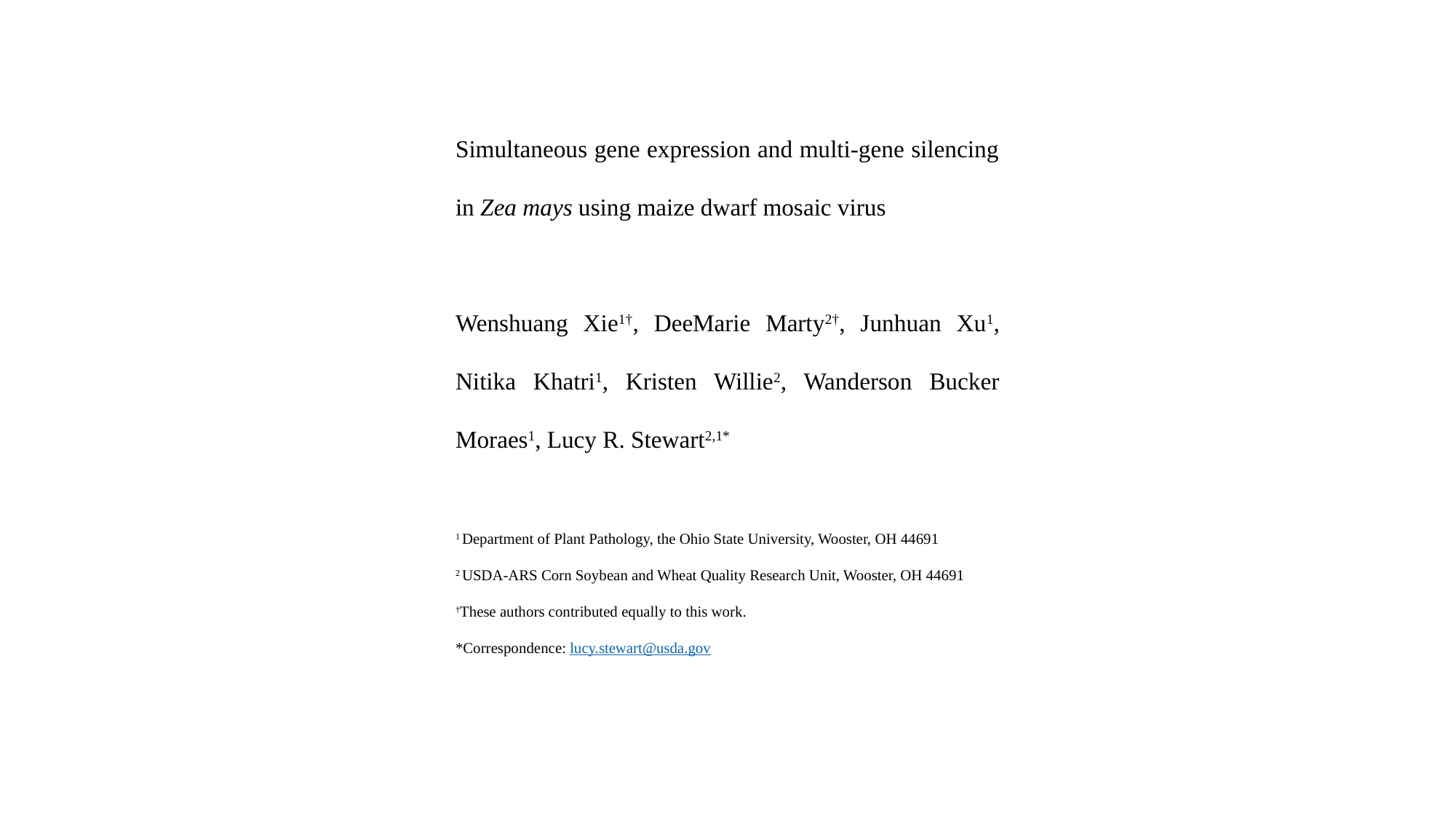

Simultaneous gene expression and multi-gene silencing in Zea mays using maize dwarf mosaic virus
Wenshuang Xie1†, DeeMarie Marty2†, Junhuan Xu1, Nitika Khatri1, Kristen Willie2, Wanderson Bucker Moraes1, Lucy R. Stewart2,1*
1 Department of Plant Pathology, the Ohio State University, Wooster, OH 44691
2 USDA-ARS Corn Soybean and Wheat Quality Research Unit, Wooster, OH 44691
†These authors contributed equally to this work.
*Correspondence: lucy.stewart@usda.gov

## Slide 2
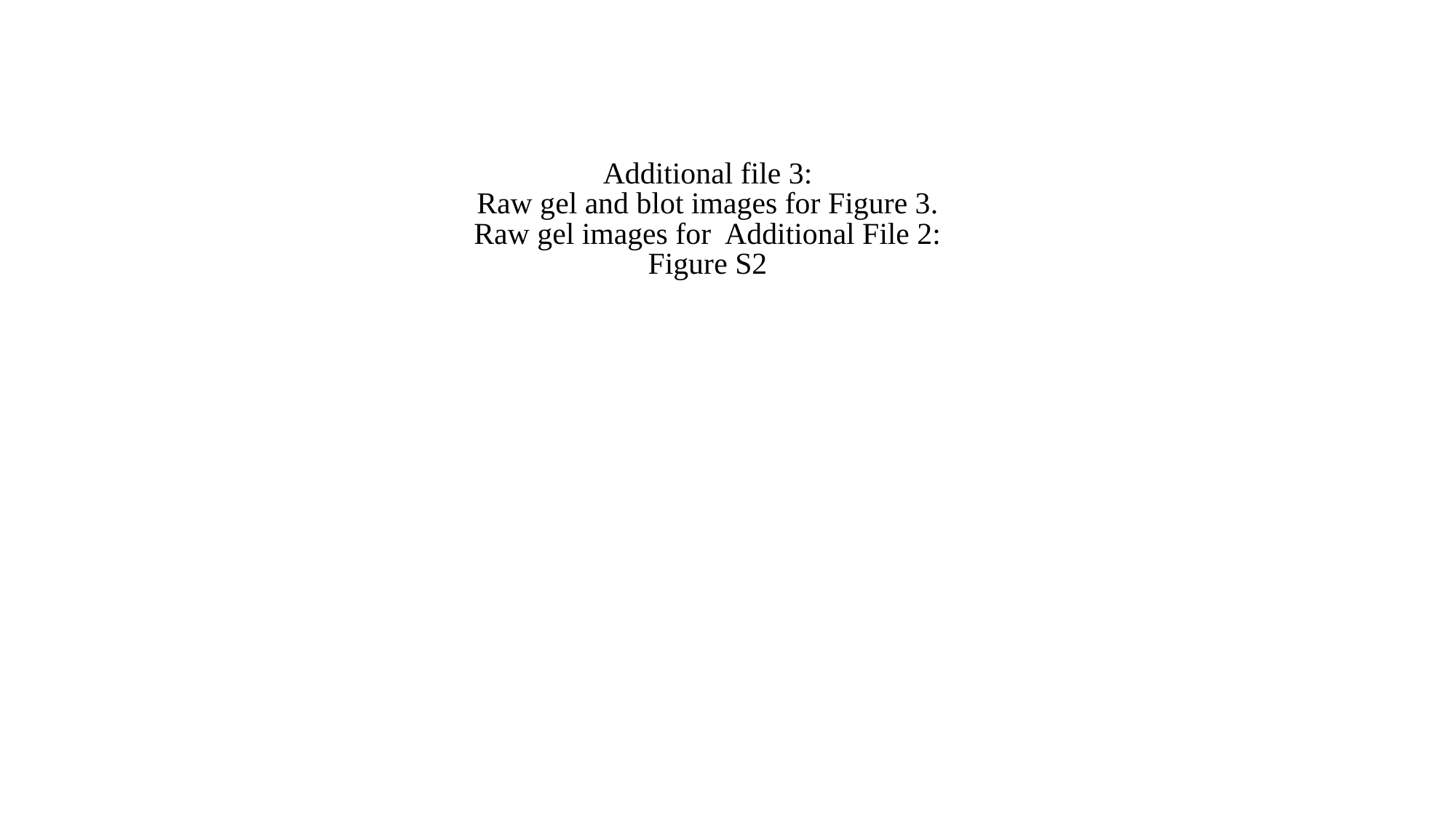

Additional file 3:
Raw gel and blot images for Figure 3.
Raw gel images for Additional File 2: Figure S2

## Slide 3
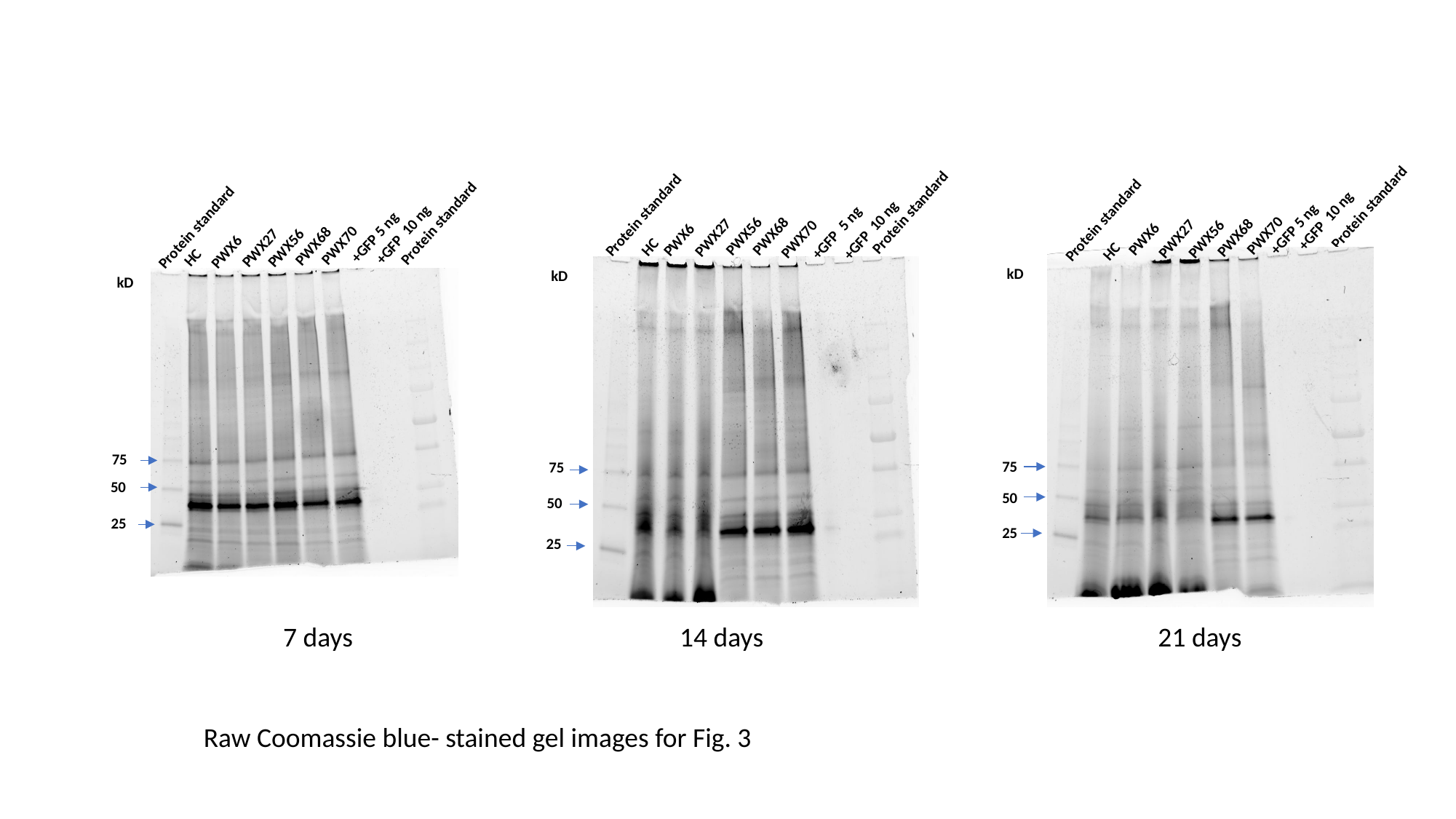

Protein standard
Protein standard
Protein standard
PWX6
Protein standard
+GFP 10 ng
Protein standard
+GFP 5 ng
+GFP 10 ng
HC
+GFP 5 ng
PWX6
+GFP 10 ng
+GFP 5 ng
PWX56
PWX68
PWX70
PWX27
PWX70
PWX6
PWX56
PWX68
PWX27
HC
PWX70
PWX68
PWX27
PWX56
HC
kD
kD
kD
75
75
75
50
50
50
25
25
25
 7 days 14 days 21 days
Protein standard
Raw Coomassie blue- stained gel images for Fig. 3

## Slide 4
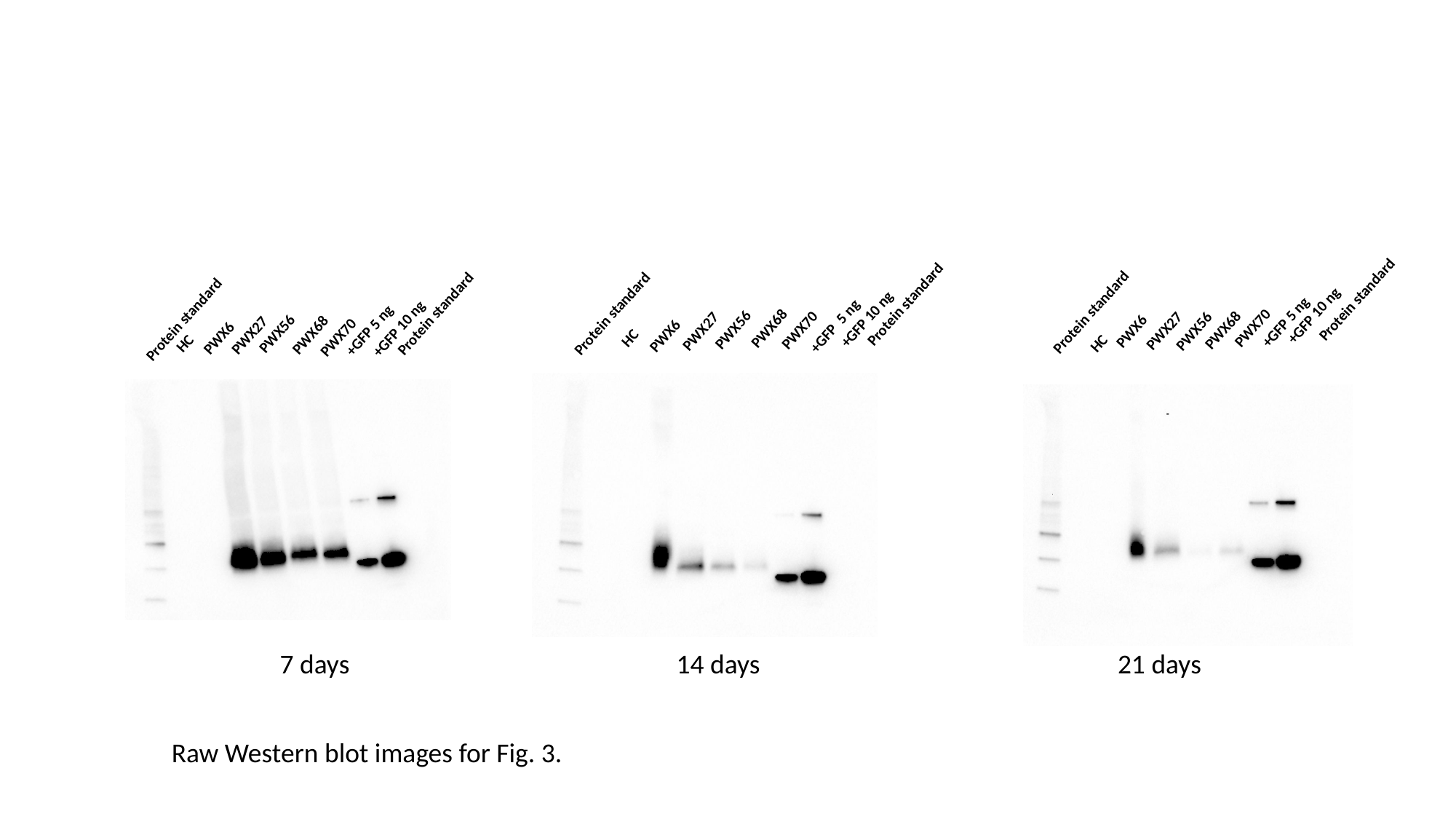

Protein standard
Protein standard
+GFP 10 ng
Protein standard
Protein standard
Protein standard
+GFP 5 ng
+GFP 5 ng
+GFP 10 ng
PWX6
Protein standard
HC
PWX6
+GFP 10 ng
+GFP 5 ng
PWX68
PWX56
PWX70
PWX70
PWX6
PWX27
PWX56
PWX68
PWX27
HC
PWX56
PWX27
PWX68
PWX70
HC
 7 days 14 days 21 days
Raw Western blot images for Fig. 3.

## Slide 5
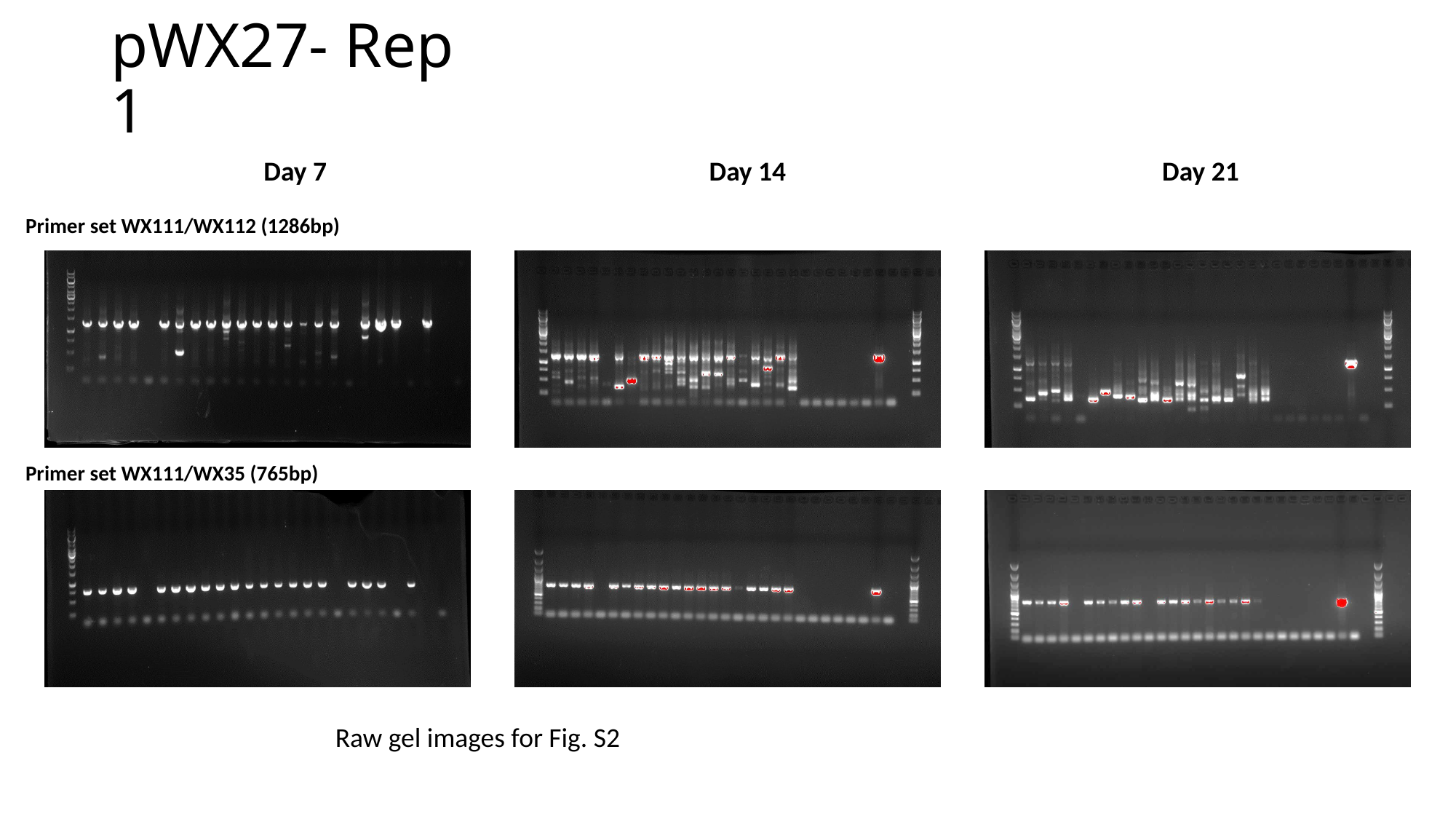

# pWX27- Rep 1
 Day 7 Day 14 Day 21
Primer set WX111/WX112 (1286bp)
Primer set WX111/WX35 (765bp)
Raw gel images for Fig. S2

## Slide 6
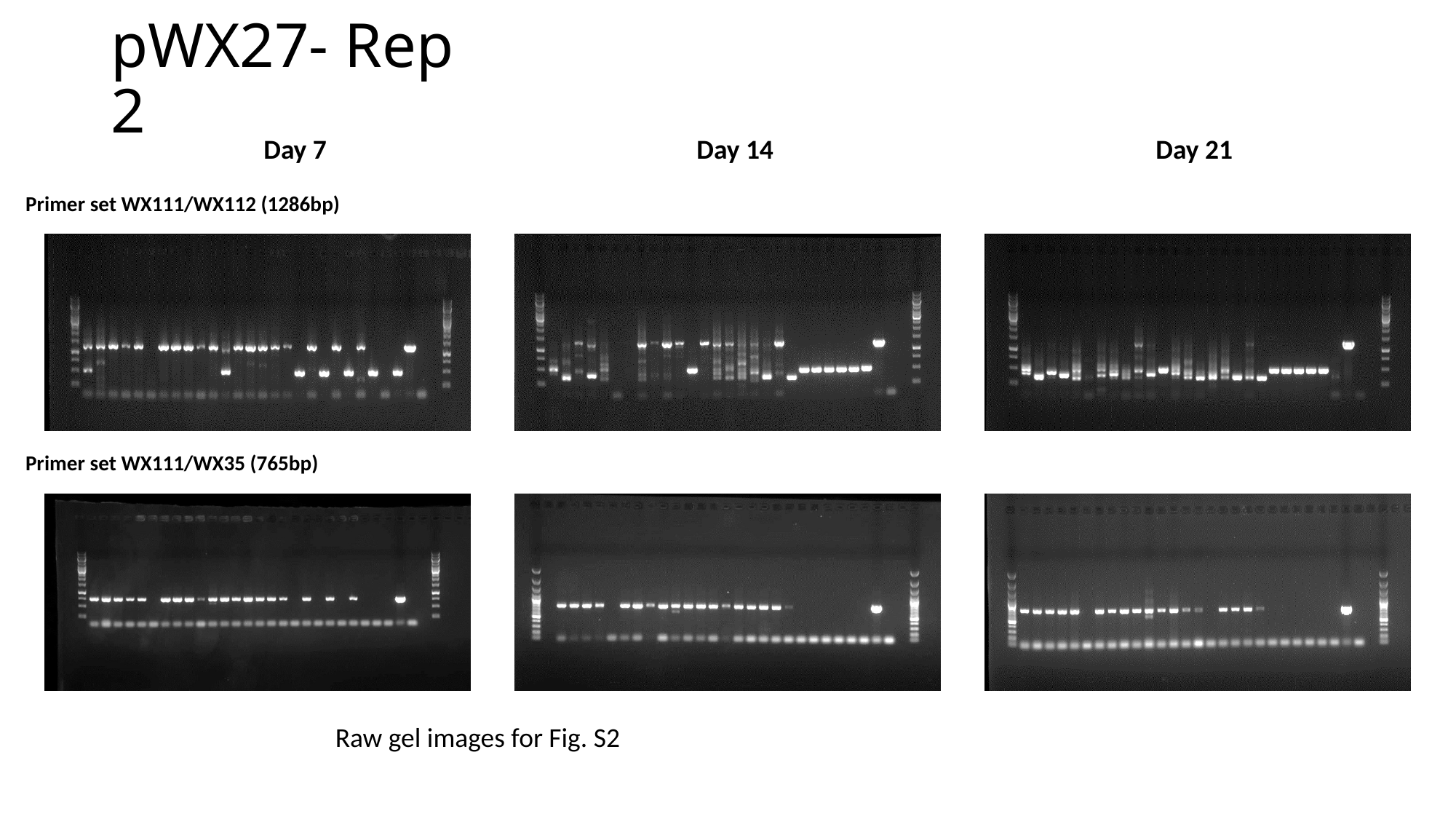

# pWX27- Rep 2
 Day 7 Day 14 Day 21
Primer set WX111/WX112 (1286bp)
Primer set WX111/WX35 (765bp)
Raw gel images for Fig. S2

## Slide 7
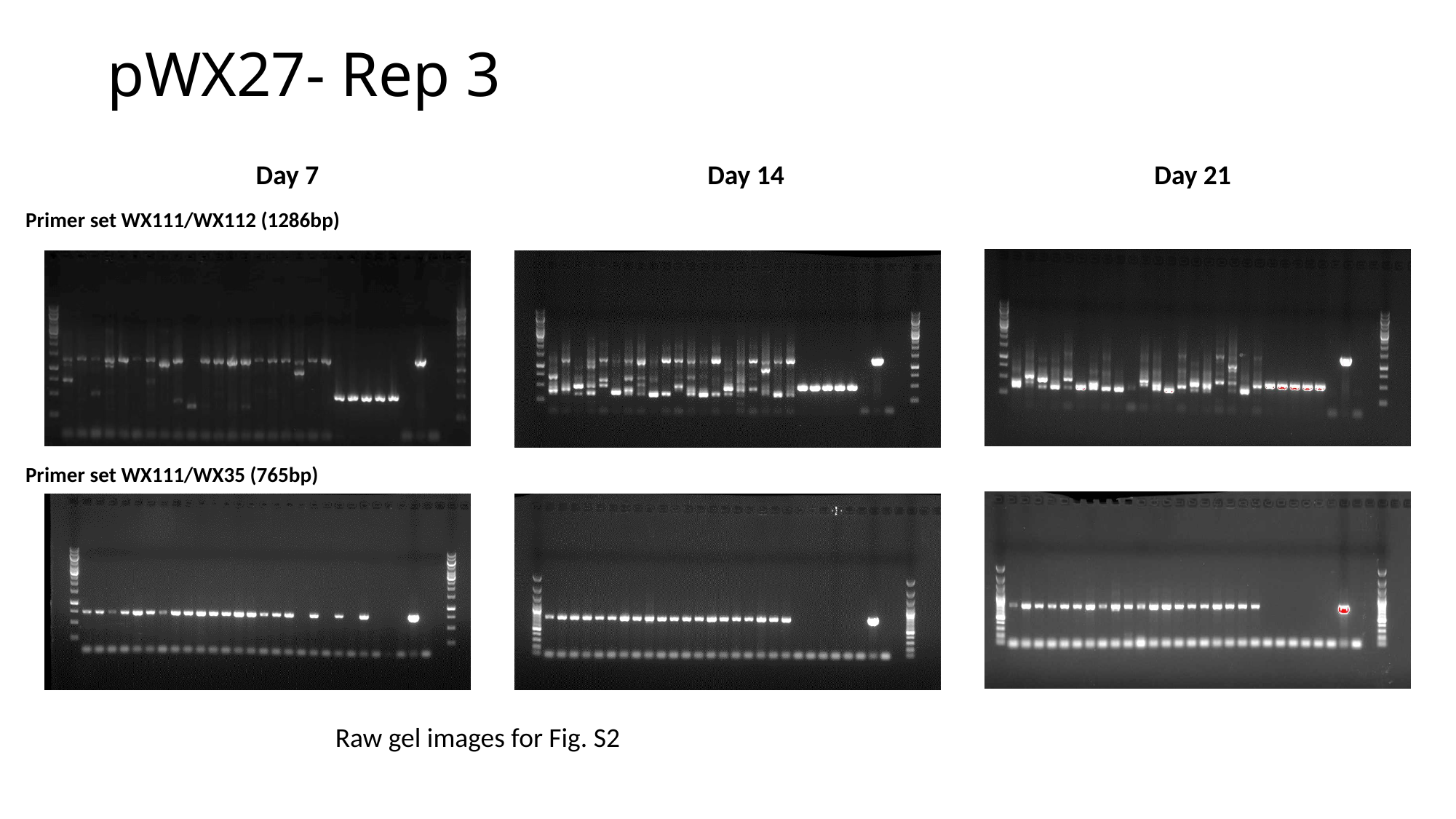

# pWX27- Rep 3
 Day 7 Day 14 Day 21
Primer set WX111/WX112 (1286bp)
Primer set WX111/WX35 (765bp)
Raw gel images for Fig. S2

## Slide 8
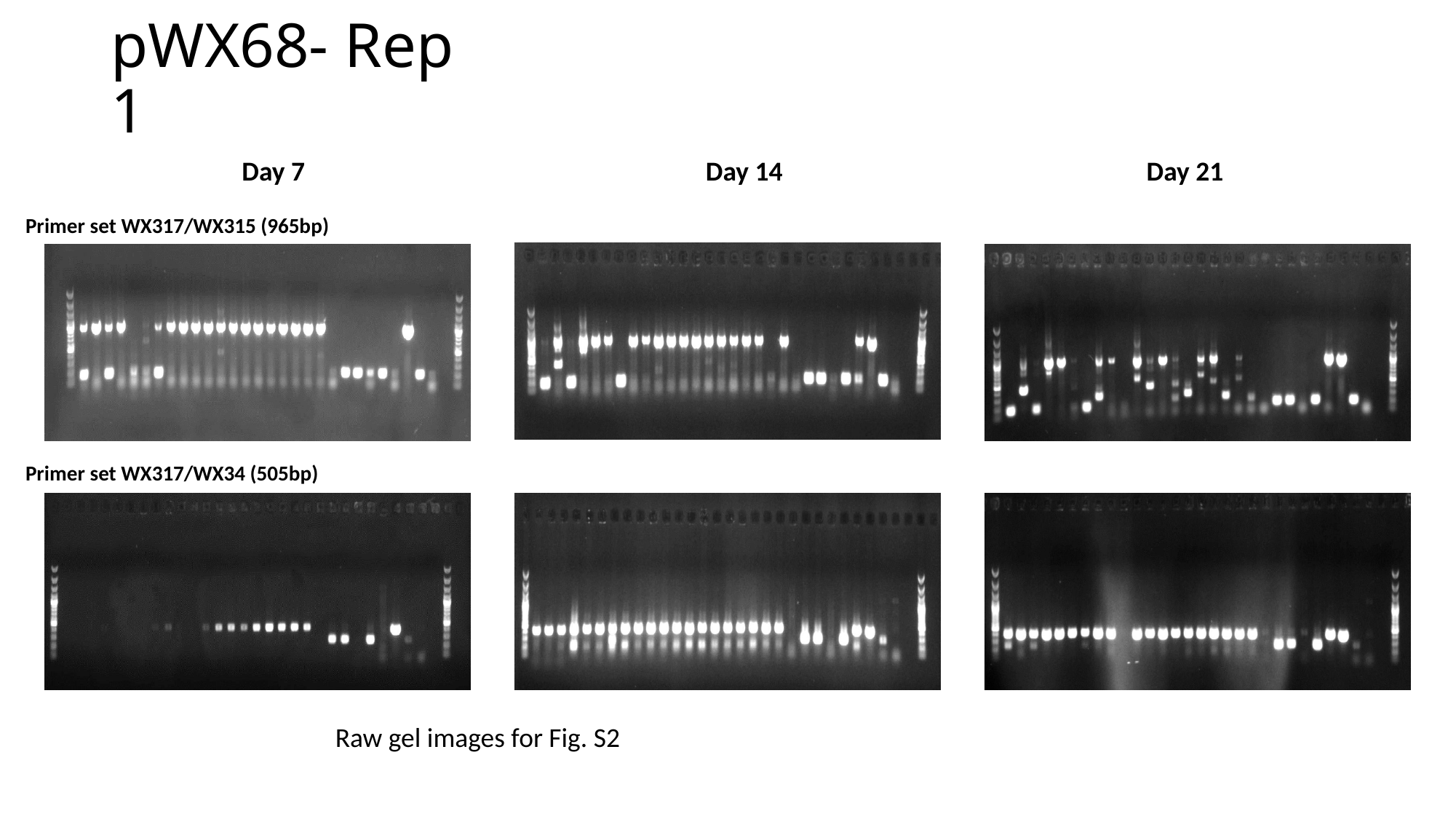

# pWX68- Rep 1
 Day 7 Day 14 Day 21
Primer set WX317/WX315 (965bp)
Primer set WX317/WX34 (505bp)
Raw gel images for Fig. S2

## Slide 9
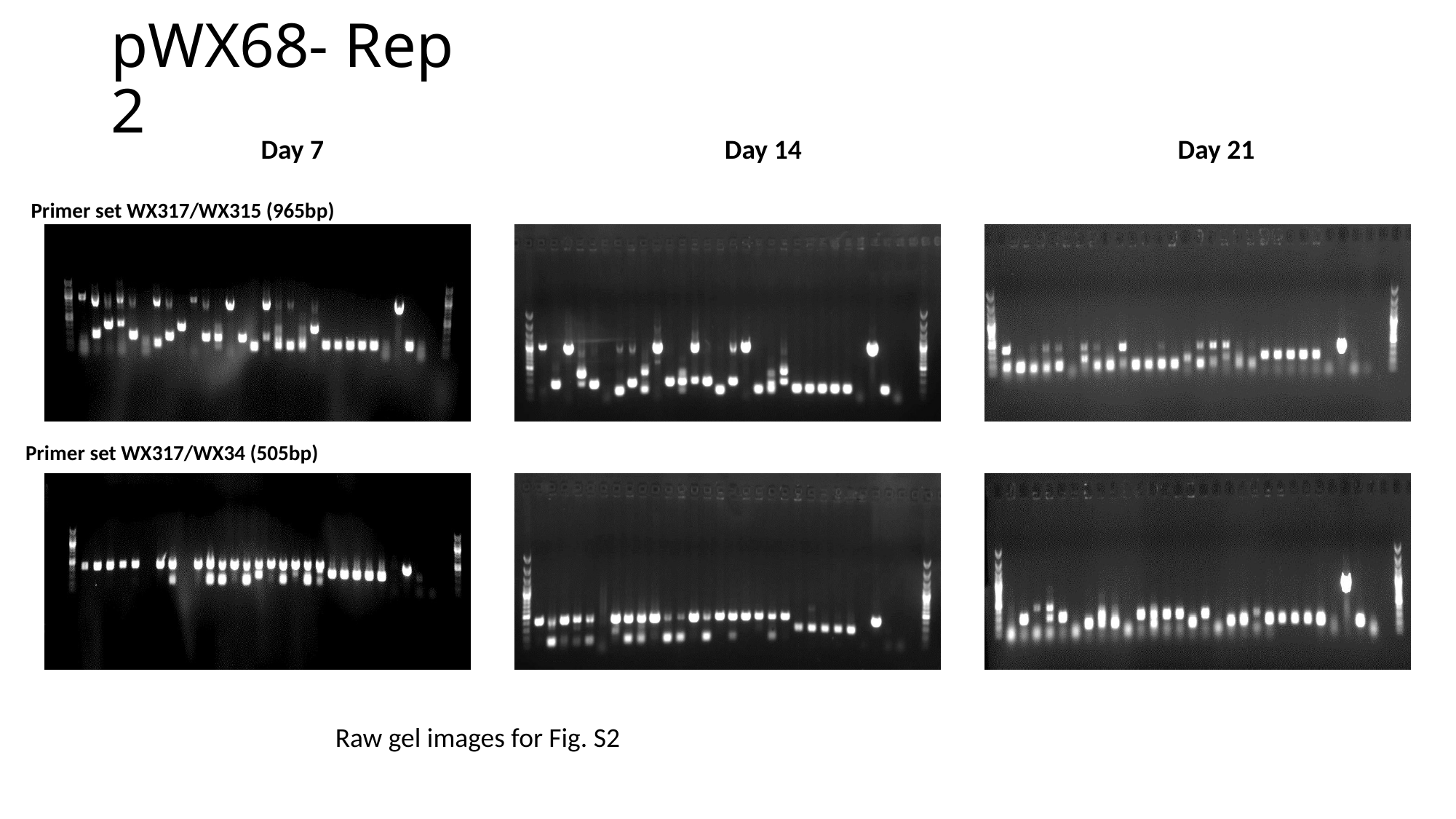

# pWX68- Rep 2
 Day 7 Day 14 Day 21
Primer set WX317/WX315 (965bp)
Primer set WX317/WX34 (505bp)
Raw gel images for Fig. S2

## Slide 10
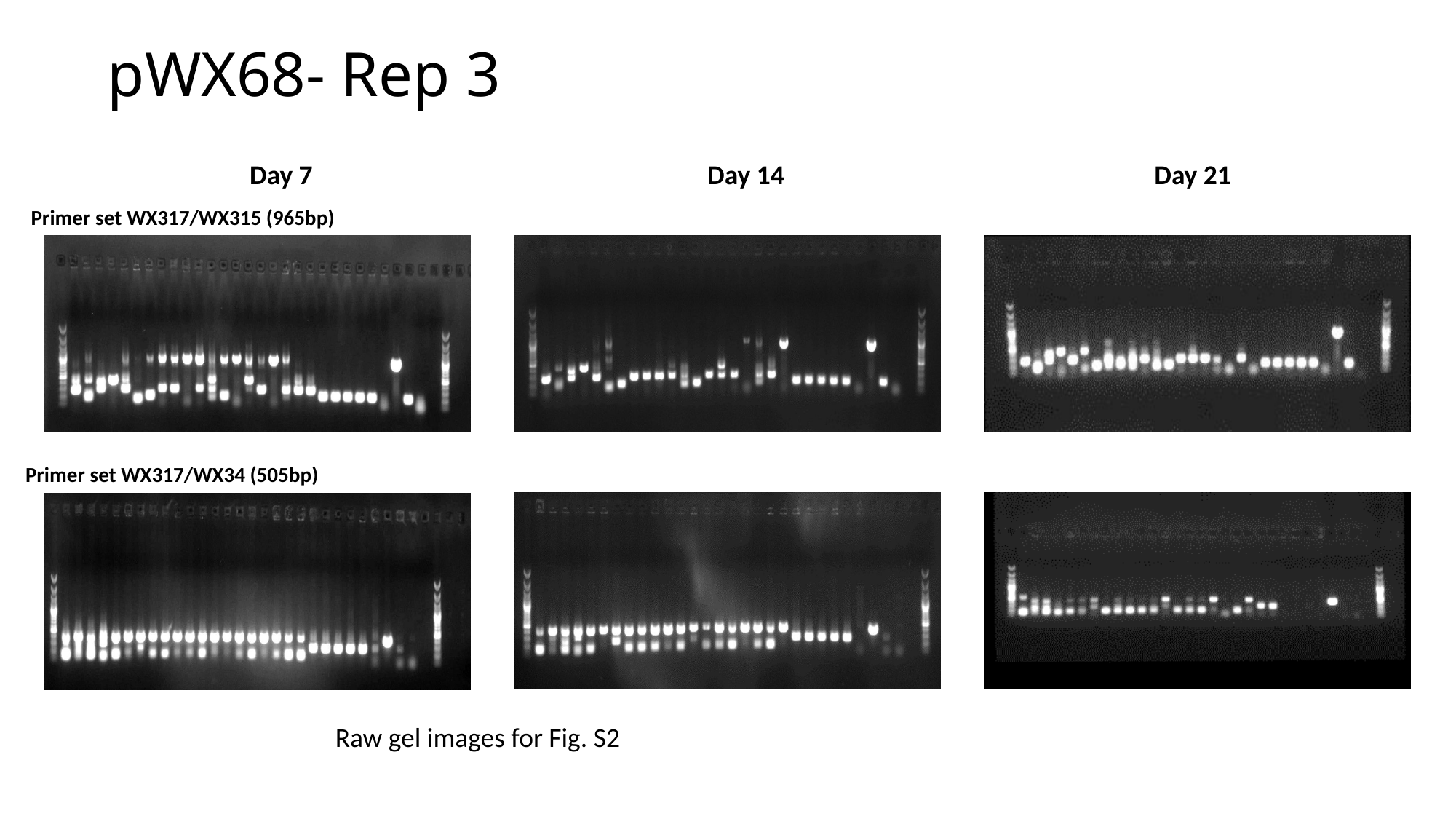

# pWX68- Rep 3
 Day 7 Day 14 Day 21
Primer set WX317/WX315 (965bp)
Primer set WX317/WX34 (505bp)
Raw gel images for Fig. S2

## Slide 11
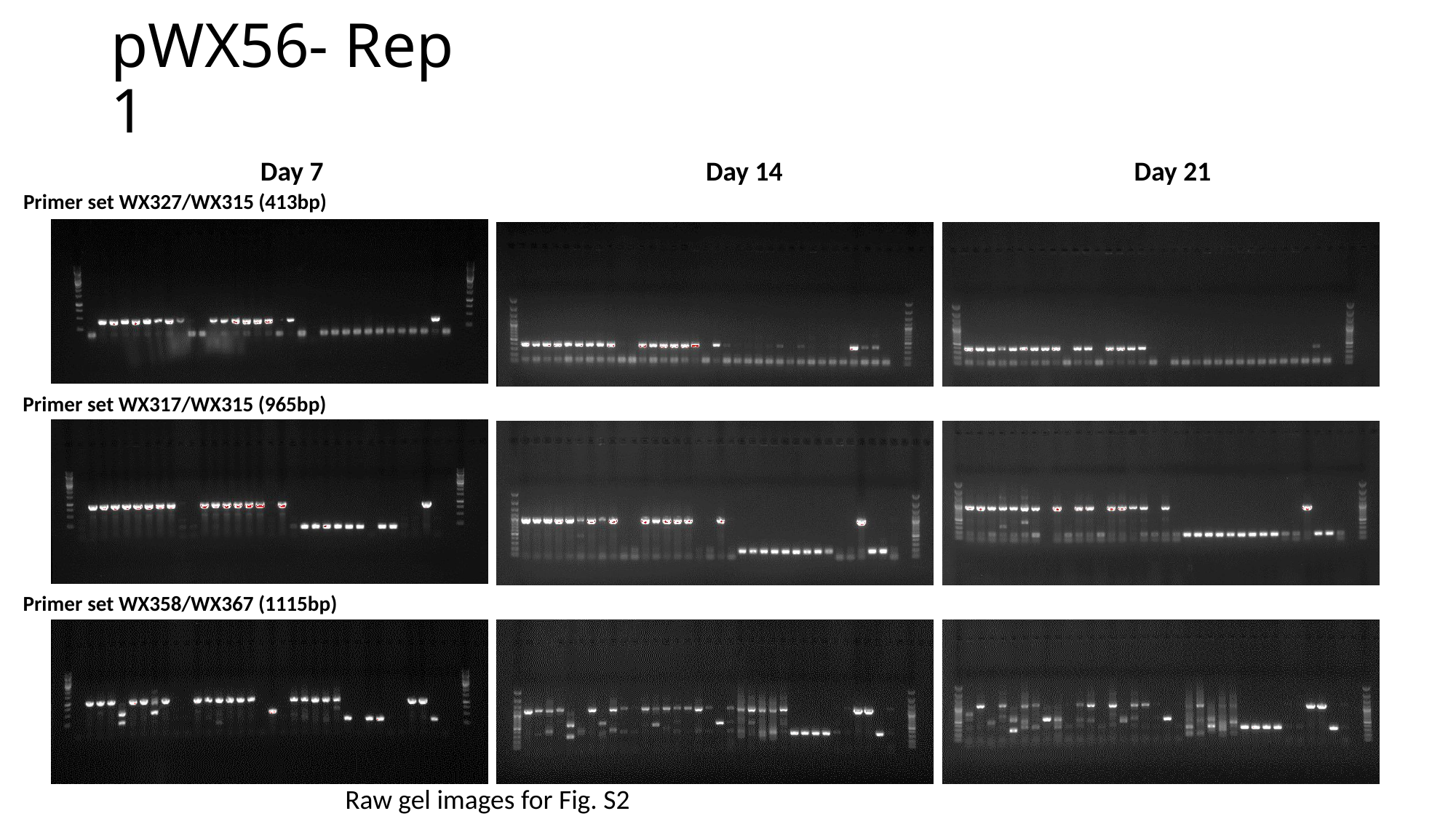

# pWX56- Rep 1
 Day 7 Day 14 Day 21
Primer set WX327/WX315 (413bp)
Primer set WX317/WX315 (965bp)
Primer set WX358/WX367 (1115bp)
Raw gel images for Fig. S2

## Slide 12
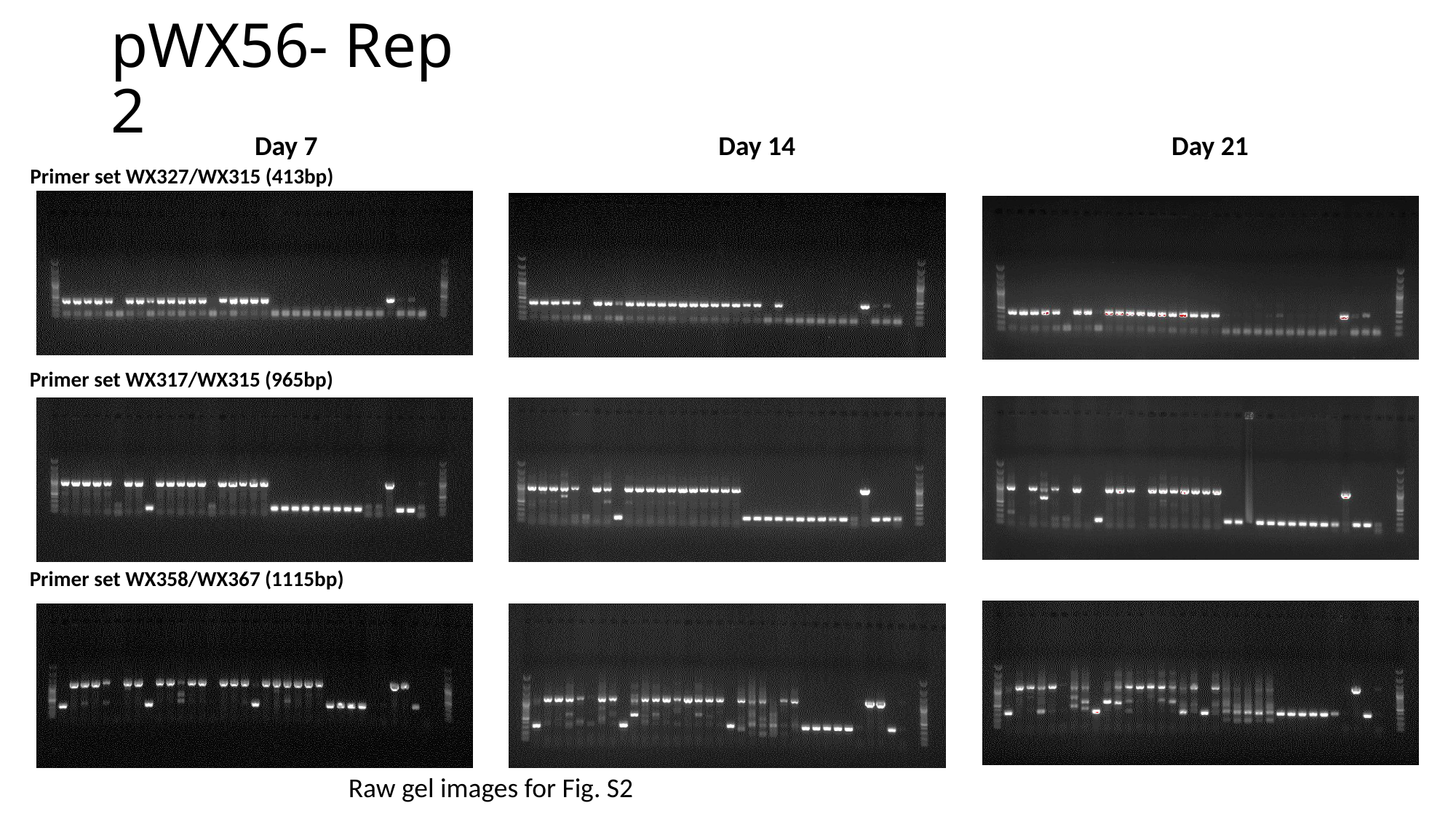

# pWX56- Rep 2
 Day 7 Day 14 Day 21
Primer set WX327/WX315 (413bp)
Primer set WX317/WX315 (965bp)
Primer set WX358/WX367 (1115bp)
Raw gel images for Fig. S2

## Slide 13
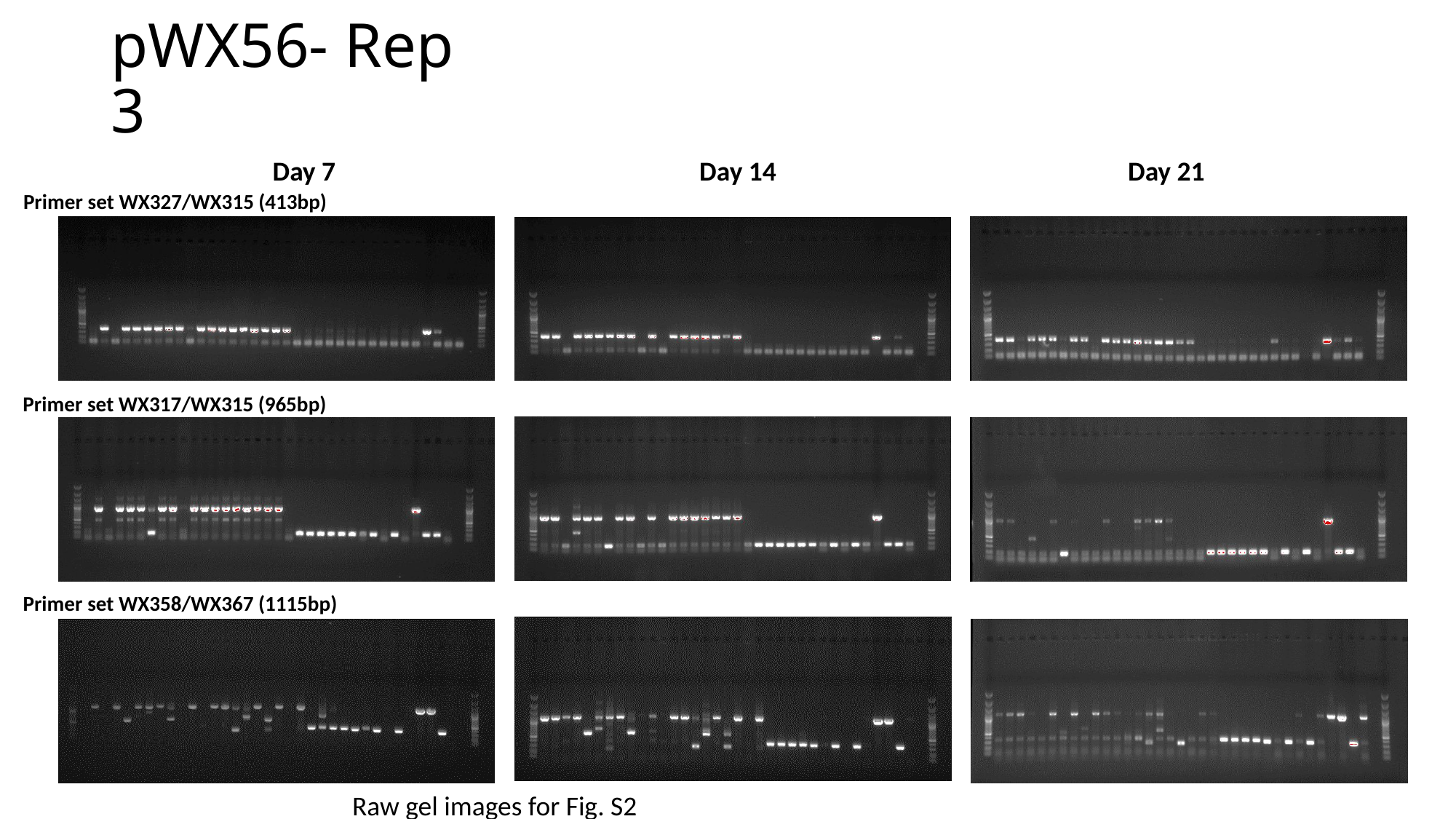

# pWX56- Rep 3
 Day 7 Day 14 Day 21
Primer set WX327/WX315 (413bp)
Primer set WX317/WX315 (965bp)
Primer set WX358/WX367 (1115bp)
Raw gel images for Fig. S2
